# Combining functional metagenomics and glycoanalytics to identify enzymes that facilitate structural characterization of sulfated *N*-glycans

**DOI:** 10.1186/s12934-021-01652-w

**Published:** 2021-08-21

**Authors:** Léa Chuzel, Samantha L. Fossa, Madison L. Boisvert, Samanta Cajic, René Hennig, Mehul B. Ganatra, Udo Reichl, Erdmann Rapp, Christopher H. Taron

**Affiliations:** 1 Bioprocess Engineering, Max Planck Institute for Dynamics of Complex Technical Systems, 39106 Magdeburg, Germany; 2 New England Biolabs, Ipswich, MA 01938 USA; 3 glyXera GmbH, 39120 Magdeburg, Germany; 4 Chair of Bioprocess Engineering, Otto-von-Guericke University, 39106 Magdeburg, Germany

**Keywords:** Sulfatase, Functional metagenomics, Glycoanalytics, Glycan analysis, Human microbiome, Glycan sulfation, N-acetylglucosamine-6-sulfate

## Abstract

**Background:**

Sulfate modification of *N*-glycans is important for several biological functions such as clearance of pituitary hormones or immunoregulation. Yet, the prevalence of this *N*-glycan modification and its functions remain largely unexplored. Characterization of *N*-glycans bearing sulfate modifications is hampered in part by a lack of enzymes that enable site-specific detection of *N*-glycan sulfation. In this study, we used functional metagenomic screening to identify enzymes that act upon sulfated N-acetylglucosamine (GlcNAc). Using multiplexed capillary gel electrophoresis with laser-induced fluorescence detection (xCGE-LIF) -based glycoanalysis we proved their ability to act upon GlcNAc-6-SO_4_ on *N*-glycans.

**Results:**

Our screen identified a sugar-specific sulfatase that specifically removes sulfate from GlcNAc-6-SO_4_ when it is in a terminal position on an *N*-glycan. Additionally, in the absence of calcium, this sulfatase binds to the sulfated glycan but does not remove the sulfate group, suggesting it could be used for selective isolation of sulfated *N*-glycans. Further, we describe isolation of a sulfate-dependent hexosaminidase that removes intact GlcNAc-6-SO_4_ (but not asulfated GlcNAc) from a terminal position on *N*-glycans. Finally, the use of these enzymes to detect the presence of sulfated *N*-glycans by xCGE-LIF is demonstrated.

**Conclusion:**

The present study demonstrates the feasibility of using functional metagenomic screening combined with glycoanalytics to discover enzymes that act upon chemical modifications of glycans. The discovered enzymes represent new specificities that can help resolve the presence of GlcNAc-6-SO_4_ in *N*-glycan structural analyses.

**Supplementary Information:**

The online version contains supplementary material available at 10.1186/s12934-021-01652-w.

## Background

Most eukaryotic proteins are covalently modified with carbohydrates (“glycans”) at certain asparagine (*N*-linked glycans (*N*-glycans)) or serine/threonine (*O*-linked glycans (*O*-glycans)) residues. It is estimated that more than half of all mammalian proteins bear some form of glycosylation [1]. Additionally, the surface of mammalian cells may contain upwards of 10 million *N*- and *O*-linked glycans attached to extracellular proteins [1]. Glycans play many important roles in secretory protein structure and function including assisting in protein folding, trafficking, tissue distribution, and persistence in the mammalian bloodstream [2]. As such, glycosylation is considered an intrinsic part of a secretory protein’s overall structure. Glycan structure itself has wide-ranging importance in both basic biology and pharmaceutical science [2–5]. Aberrant glycosylation has been implicated in numerous diseases [6, 7] and changes in *N*-glycans of serum glycoproteins has yielded promising disease biomarkers [7–9]. Additionally, *N*-glycan composition can affect the function and efficacy of many glycoprotein drugs like certain monoclonal antibodies, growth factors and fusion proteins [10, 11], and is a critical quality attribute that is monitored during drug manufacturing.

Protein glycosylation is inherently complex due to the manner in which glycans are assembled and processed by cells. Glycan synthesis does not occur through copying or interpreting a biological template (like DNA, RNA, or protein synthesis). Instead glycans are synthesized via complex biosynthetic pathways involving numerous glycosyltransferases that assemble different monosaccharides into specific structures. There are over 200 glycosyltransferases encoded in the human genome and expression of many varies by cell type, tissue type or in response to environmental factors. This results in substantial glycan structural heterogeneity. For example, over 140 different *N*-glycan structures have been identified on glycoproteins present in human serum [12]. Adding to this complexity, glycans can also be modified with sulfate, phosphate, methyl or acetyl groups on certain sugars. These groups are collectively termed ‘post-glycosylation modifications’ (PGMs) and each imparts a variety of biological functions (See [13] for a review).

**Fig. 1:**
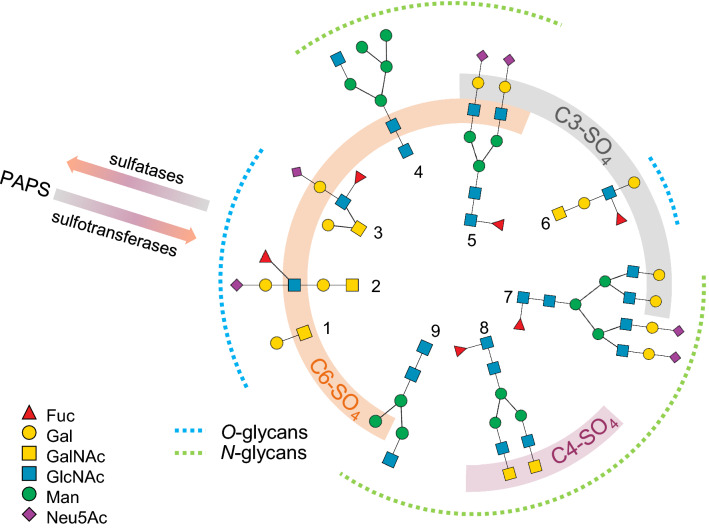
*N*- and *O*-glycan sulfation. *N*- and *O*-glycans can be chemically modified with sulfate. Sulfation typically occurs at position C6 on GlcNAc, GalNAc, Gal or Man (orange highlight), at position C4 on GalNAc (grey highlight) or at position C3 on Gal (purple highlight). Examples of sulfated *O*-glycans from human glycoproteins (1, 2, 3 and 6) [15–18], and sulfated *N*-glycan structures from Influenza virus (4), human peripheral blood cells (5), human Tamm-Horsfall glycoprotein (7), human pituitary hormones (8) [14] and *Panulirus interruptus* hemocyanin (9) [19] are shown. Glycans are represented using SNFG nomenclature [69]

One major type of PGM involves enzymatic modification of glycans with sulfate. Sulfation has been observed on all classes of glycans, including *N*- and *O*-glycans. Sulfation occurs in the Golgi where a sulfate group is transferred from 3′-phosphoadenosine-5′-phosphosulfate (PAPS) to specific positions on sugars by sulfotransferases. Sulfation of N-acetylglucosamine (GlcNAc-6-SO_4_), N-acetylgalactosamine (GalNAc-6-SO_4_ and GalNAc-4-SO_4_), galactose (Gal-3-SO_4_ and Gal-6-SO_4_) and mannose (Man-6-SO_4_) has been observed in *N*- and *O*-glycans [14–20] (Fig. 1). Sulfation of *N*- and *O*-glycans has been implicated in many biological processes. Sulfated *N*-glycans are prevalent on both influenza virus A neuraminidase and hemagglutinin surface proteins and likely play a role in virus replication and virulence [14, 21]. Additionally, a GlcNAc-6-SO_4_ α2-6-sialyl LacNAc *N*-glycan epitope on B lymphocytes is a preferred ligand for the CD22 receptor, indicating a role in immunoregulation [13]. Recently, sulfated *N*-glycans present on serum immunoglobulin G were proposed as potential biomarkers for rheumatoid arthritis [22].

The study of sulfated *N*- and *O*-glycans can be technically challenging and better tools are needed to aid their analysis. Structural characterization of sulfated glycans has been accomplished using mass spectrometry (e.g., LC–MS/MS or MALDI-TOF) of released glycans or glycopeptides [14, 22–25]. These methods can be compromised by poor ionization of sulfated species and the natural low abundance of sulfated *N*- and *O*-glycans. Methods to enrich samples for sulfated glycans could aid their analysis. Glycan-specific binding proteins have recently been used to isolate glycans possessing certain structural features from complex samples [26–30]. However, no sugar sulfate-specific binding proteins have yet been applied to enrich sulfated glycans. Additional analytical methods involving multiplexed capillary gel electrophoresis with laser-induced fluorescence detection (xCGE-LIF) [31–33] and high/ultra-performance liquid chromatography coupled to fluorescence detector (H/UPLC-FLR) methods [34–37] are also routinely used to separate glycans and match their migration- and retention-times, respectively, to those of known glycans in reference databases. These methods of glycan analysis often use highly specific exoglycosidases to enzymatically confirm the presence or absence of certain sugars [38–40]. However, enzymes that remove site-specific sugar sulfation or sulfated sugars are currently missing from the glycoanalysis toolbox.

In this study, we sought to improve the range of analytical enzymes available for analysis of sulfated glycans. We successfully applied high-throughput functional metagenomic screening to identify enzymes that manipulate GlcNAc-6-SO_4_, a sulfated sugar found in some *O*-glycans and certain mammalian *N*-glycan outer arms. We devised a novel coupled screening assay using a fluorescent GlcNAc-6-SO_4_ analog and an exogenous hexosaminidase. In this assay the coupling enzyme can act upon the substrate and release the fluorophore only if the sulfate group was first hydrolyzed by an enzyme expressed from a metagenomic clone. Our screen identified a sulfatase and hexosaminidase that each act exclusively upon GlcNAc-6-SO_4_. We further employed xCGE-LIF-based glycoanalysis to evaluate these enzymes activity on *N*-glycan substrates and showed their suitability for structural characterization and enrichment of *N*-glycans bearing terminal GlcNAc-6-SO_4_.

## Results

### Functional screening for sulfatases

A fosmid library containing large DNA inserts (~ 40 kb) from human gut microbiota was created in *E. coli*. The library diversity was assessed by analyzing cloned fragments from 24 randomly picked transformants. Sequencing of fosmid inserts revealed DNA originating from various enteric bacteria species such as *Bifidobacterium longum* (3 clones), *Bifidobacterium adolescentis* (4 clones), *Bacteroides sp.* (2 clones), *Phocaeicola dorei* (2 clones), *Faecalibacterium prausnitzii* (1 clone), *Adlercreutzia sp.* (2 clones) and *Collinsella aerofaciens* (1 clone). All identified species belonged to the phyla Bacteroides, Actinobacteria or Firmicutes, consistent with the composition of a healthy individual’s gut microbiota [41]. Interestingly, 4 clones (> 15% of those analyzed) contained insert DNA from unknown or un-sequenced species. For 5 clones, the quality of the Sanger sequencing data did not permit a conclusion about the origin of the cloned DNA. In a second investigation, 12 clones were subjected to restriction analysis using the endonuclease FseI. Distinct restriction patterns were observed, indicating that each clone possessed a unique cloned DNA fragment (Additional file 1: Fig. S1). The average insert size was estimated to be 30–40 kb. No empty fosmid vector was observed. The library quality both in terms of genetic diversity and insert size was deemed suitable for function-based enzyme screening. As such, a collection of 23,040 clones were individually archived for use in various screens.

**Fig. 2:**
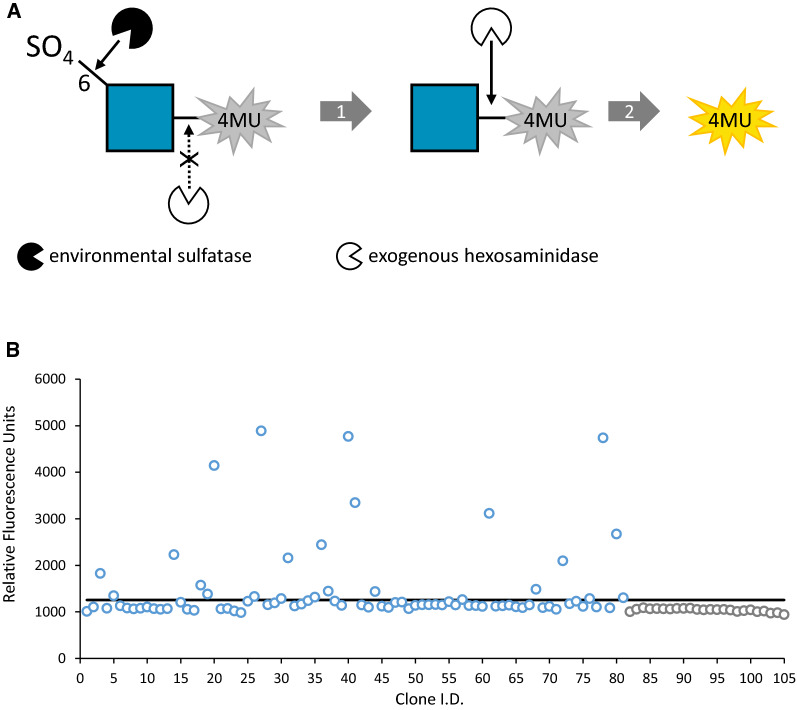
Screening a human gut metagenomic library for sulfatases. **A** Coupled assay for sulfatase screening. The substrate consists of a GlcNAc residue linked to the fluorophore 4-methylumbelliferone (4MU). The GlcNAc molecule is modified at carbon 6 with a sulfate group (SNFG notation, GlcNAc, blue square). In a first reaction, a sulfatase expressed from a metagenomic clone removes the C6 sulfate. In a second reaction, an exogenous hexosaminidase (that is inactive on the sulfated substrate) liberates the fluorophore from GlcNAc, generating a fluorescence signal. **B** Re-screening human gut metagenomic clones for sulfatase activity. Represented are fluorescence values for the 30 h timepoint. Values above the mean + 6σ (black line) were considered ‘hits’. Control lysates from clones carrying an empty pCC1 fosmid (grey circles) were assayed along with the metagenomic clones (blue circles) from the primary screen

In this study, we sought to identify enzymes proficient at removing sulfate from the 6-carbon (C6) of GlcNAc. A coupled assay using the substrate 4-methylumbelliferyl N-acetyl-β-d-glucosaminide-6-sulfate (4MU-GlcNAc-6-SO_4_) and an exogenous GlcNAc-6-SO_4_-resistant hexosaminidase was devised (Fig. 2A). In this assay, the activity of a sulfatase alone is not sufficient to release the 4MU fluorophore. Thus, the assay contains an exogenous hexosaminidase that is blocked by the presence of sulfate at the C6 position of GlcNAc. This enzyme enables fluorescence generation only if the C6-sulfate has been removed by a cloned environmental sulfatase. It is important to note that this screening strategy will also identify hexosaminidases that are able to directly hydrolyze C6-sulfated GlcNAc from 4MU.

A primary screen was conducted using the 4MU-GlcNAc-6-SO_4_/hexosaminidase coupled assay with 11,520 cell lysates (half the library) comprising the human gut metagenomic DNA clone collection (see “Methods”). A total of 81 “hits” were identified by measuring an increase in fluorescence over time. A hit was defined as a fluorescence reading 3 standard deviations over the mean background fluorescence value in at least one measured time point. This definition was intentionally liberal to capture all potential hits. The 81 hits were then re-screened using the same assay, but with a more stringent hit definition consisting of a fluorescence reading over 6 standard deviations from the mean control background fluorescence in at least two timepoints. This secondary analysis yielded 24 hits (Fig. 2B) indicating an overall screen hit rate of 0.2%.

Enzyme activity in lysates from these 24 clones was then assessed using different substrates: i) 4MU-SO_4_ to detect general sulfatase activity, ii) 4MU-GlcNAc (no sulfate) to detect hexosaminidase activity, and iii) 4MU-GlcNAc-6-SO_4_ (without exogenous hexosaminidase) to detect the activity of hexosaminidases that are not inhibited by the sulfate moiety. Twenty out of the 24 hits showed activity on 4MU-GlcNAc-6-SO_4_ in absence of exogenous hexosaminidase while 11 of these also retained activity on non-sulfated GlcNAc. These data suggest that most fosmids likely encoded a combination of both hexosaminidases and sulfatases. Only 6 hits were active on the general sulfatase substrate 4MU-SO_4_, suggesting the presence of a sulfatase that does not exclusively recognize GlcNAc-6-SO_4_, and implying that the observed sulfatases from other clones may be sugar specific-sulfatases that strictly hydrolyze sulfate located on a sugar ring. These biochemical observations were further reconciled with nucleotide sequencing of fosmid inserts.

### Analysis of fosmid DNA sequences

Two multiplexed Pacific Bioscience (PacBio) libraries were constructed to sequence fosmids isolated from all 24 clones. Nineteen were successfully assembled in single contigs with insert sizes of 30–45 kb. These nucleotide sequences termed F1-F19 were deposited to GenBank under the accession numbers MW677166-MW677184. Five fosmids could not be sequenced properly with attempted assemblies resulting in multiple contigs or failing due to a lack of sequence coverage. Contamination of the fosmid preparation with *E. coli* genomic DNA, the presence of long repeats, or a co-culture of two distinct clones in the same well of the 384 well plate may be responsible for the failed assemblies [42]. As such, these 5 clones were not further analyzed.

Open reading frames (ORFs) encoded by each of the remaining 19 clones were predicted using MetaGeneMark. For each ORF, a MegaBLAST search against the NCBI protein repository was performed, and ORF maps for each of the 19 inserts were drawn (Additional file 1: Fig. S2). For two clones (F13 and F15), genes constituting the riboflavin biosynthesis pathway (ribD, ribE, ribB/A and ribH) were observed (Additional file 1: Fig. S2N and S2P) [43]. Multiple metabolites produced by this pathway, including riboflavin and luminazine, are fluorescent compounds having excitation/emission spectra that partially overlap with that of 4MU [44, 45]. These clones were also fluorescent in our assay in the absence of the 4MU-GlcNAc-6-SO_4_ substrate (Additional file 1: Fig. S3), indicating they were false positives. It is noteworthy that the riboflavin synthesis pathway has been consistently identified in other screens conducted in our laboratory where 4MU substrates have been used. Sequence analysis also showed that 5 clones (F14, F16, F17, F18, and F19) did not encode obvious sulfatase, hexosaminidase, or fluorescent metabolite producing pathways (Additional file 1: Fig. S2O, S2Q, S2R, S2S, S2T). However, these five clones had very low fluorescence signals in secondary screening. As such, 7 fosmids (F13–F19) were dismissed from this initial study and the remaining 12 fosmids (F1–F12) were further analyzed.

**Fig. 3:**
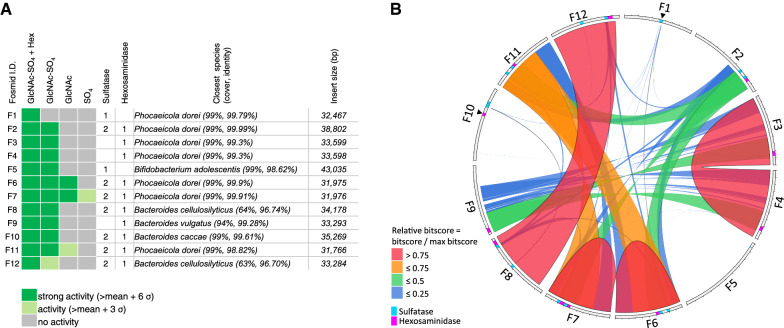
Function- and sequence-based analysis of fosmids F1-F12. **A** Rescreening analysis of fosmids F1-F12. Fosmids F1-F12 were tested using four distinct assays: using 4MU-GlcNAc-6-SO_4_ with exogenous hexosaminidase (GlcNAc-SO_4_ + Hex), 4MU-GlcNAc-6-SO_4_ in the absence of exogenous enzyme (GlcNAc-SO_4_), asulfated 4MU-GlcNAc (GlcNAc), and 4MU-SO_4_ (SO_4_). For each fosmid, shown is its activity in each assay, the predicted number of sulfatase and hexosaminidase genes found within its DNA sequence, the closest species of origin as determined by BLAST against the entire NCBI nucleotide collection, and the size of the cloned insert. **B** Sequence alignment of fosmids F1-F12. A Circos plot made with Circoletto software illustrates relatedness in the DNA sequences of the 12 clones. Ribbons are colored using BLAST bitscores within Circoletto

The sequences of fosmids F1-F12 were assessed for the presence of sulfatases and hexosaminidases from known protein families (Fig. 3A). In total, 16 sulfatase genes encoded by 9 of the 12 clones were identified by homology, with 7 clones each encoding two putative sulfatases. Interestingly, of the 9 clones having putative sulfatase genes, 7 also encoded a putative hexosaminidase. Overall, 10 hexosaminidases were identified (Fig. 3A). The fosmids F1-F12 were also compared to each other using Circos plots generated by Circoletto (Fig. 3B) [46]. This revealed that clones F3 and F4 both harbored the same region of the *Phocaeicola dorei* genome and were closely related to portions of F9. These three clones lacked predicted sulfatase genes but contained the same hexosaminidase gene, suggesting they might encode a protein with the ability to directly release intact GlcNAc-6-SO_4_ from 4MU-GlcNAc-6-SO_4_. Clones F8 and F12 were also highly similar fosmids containing the same genome locus of a species related to *Bacteroides cellulosilyticus* with ~ 60% sequence identity for the analyzed fragments. Finally, a group of 4 highly related sequences was comprised of fosmids F6, F7, F11 and F2 (with F6 and F7 being nearly identical). Considered together, the Circos analysis showed that 8 sulfatase genes out of the 16 initially identified were distinct. The redundancy in fosmids F1–F12 suggests that the number of clones we screened was sufficient to explore the full diversity of the library. Thus, the 8 distinct sulfatase genes and the hexosaminidase gene (common to fosmids F3, F4 and F9) were selected for further biochemical exploration.

### Identifying genes encoding active sulfatases using in vitro protein expression

**Fig. 4:**
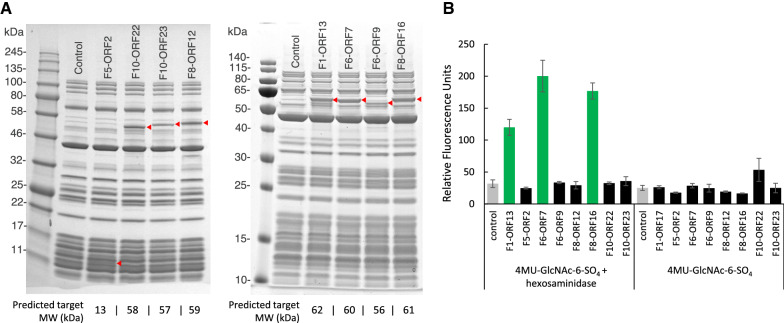
In vitro expression of sulfatase candidates. **A** SDS-PAGE of 8 sulfatases produced with the PURExpress® system. Expressed enzymes are shown with a red triangle. **B** Activity of in vitro expressed sulfatases. Sulfatases were assayed on 4MU-GlcNAc-6-SO_4_ in the presence and absence of exogenous hexosaminidase

To rapidly test candidate sulfatase genes for activity, each was expressed in vitro using the PURExpress® in vitro transcription/translation system and assayed for activity (Fig. 4). Each of the 8 sulfatase genes expressed protein of the expected size (Fig. 4A). Of these, three proteins (F1-ORF13, F6-ORF7 and F8-ORF16) also showed activity on 4MU-GlcNAc-6-SO_4_ supplemented with hexosaminidase_,_ but showed no activity in control reactions lacking hexosaminidase (Fig. 4B). Interestingly, F10 encoded two putative sulfatases (F10-ORF22 and F10-ORF23) but neither showed statistically significant activity (Fig. 4B).

### Protein sequence analysis of active sulfatases

The deduced protein sequences of the 3 active sulfatases (F1-ORF13, F6-ORF7 and F8-ORF16) were compared to proteins present in GenBank. All three enzymes had strong homology to enzymes from *Phocaeicola* and *Bacteroides* species (Additional file 1: Fig. S4A). During secondary screening, clone F1 was the only clone that retained activity solely on 4MU-GlcNAc-6-SO_4_ in presence of an exogenous hexosaminidase (Additional file 1: Fig. 3A). As such, F1-ORF13 was chosen for further biochemical characterization.

### Determination of F1-ORF13 sulfatase specificity using sulfated monosaccharides

**Fig. 5:**
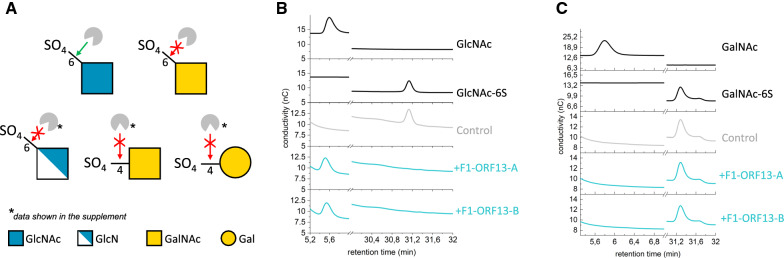
F1-ORF13 sulfatase specificity. **A** Summary of the specificity of F1-ORF13 on monosaccharide substrates. Monosaccharides are represented using SNFG nomenclature [69]. A red crossed arrow indicates no activity of F1-ORF13 on these substrates. **B**, **C** Activity of in vitro expressed F1-ORF13 sulfatase on GlcNAc-6-SO_4_ (**B**) and GalNAc-6-SO_4_ (**C**). Analysis was performed by high-performance anion-exchange chromatography with pulsed amperometric detection. Reactions were performed in duplicate (F1-ORF13-A and -B – blue chromatograms). Control reactions consists of in vitro expression in the absence of F1-ORF13 (grey chromatogram). Sulfated and non-sulfated monosaccharides dissolved in water were run as retention time standards (black chromatograms)

To determine the sulfate specificity of F1-ORF13, its activity on the sulfated monosaccharides GlcNAc-6-SO_4_, GlcN-6-SO_4_, GalNAc-6-SO_4_, GalNAc-4-SO_4_ and Gal-4-SO_4_ was evaluated (Fig. 5 and Additional file 1: Fig. S5). Sulfated monosaccharides and their corresponding asulfated forms were each incubated with F1-ORF13 and reaction products were separated by high-performance anion-exchange chromatography with pulsed amperometric detection (HPAEC-PAD). Sulfate removal was only observed for GlcNAc-6-SO_4_ and no other substrates, indicating that F1-ORF13 is highly selective for sulfation of carbon-6 of GlcNAc. Furthermore, the enzyme’s inability to remove sulfate from similar monosaccharides GlcN-6-SO_4_ or GalNAc-6-SO_4_ implies the acetyl group on GlcNAc or the stereochemistry around carbon-4 are likely important determinants for substrate recognition.

### F1-ORF13 sulfatase activity on GlcNAc-6-SO_4_ in intact *N*-glycans

**Fig. 6:**
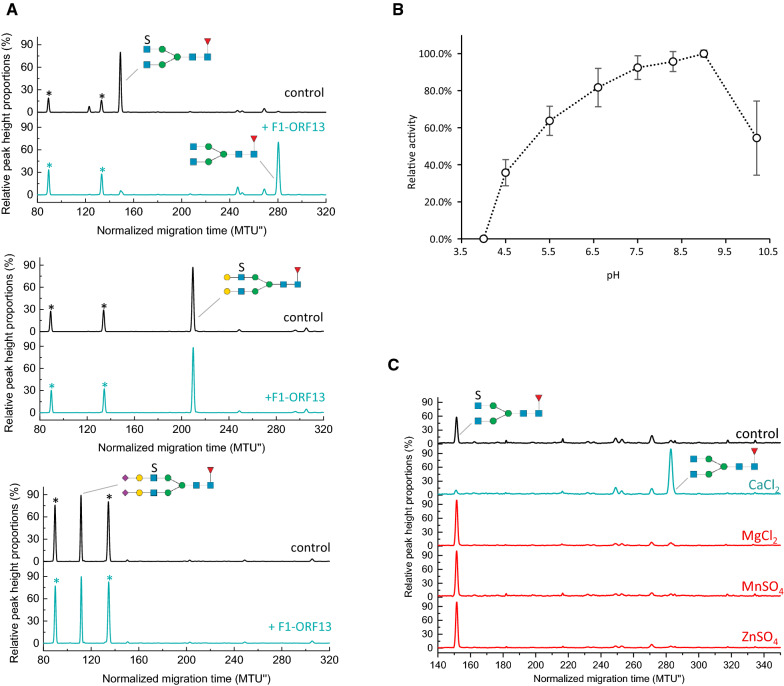
F1-ORF13 sulfatase activity on *N*-glycans. **A** F1-ORF13 sulfatase substrate specificity. F1-ORF13 was assayed on different APTS-labelled *N*-glycan substrates generated from human immunoglobulin. Substrate and product were monitored by xCGE-LIF. Structure assignment was enabled by comparing the normalized migration times with those of a *N*-glycan database and confirmed by exoglycosidase digests (Cajic S, Hennig R, Grote V, Reichl U and Rapp E; manuscript in preparation). **B** F1-ORF13 optimal pH. Optimal pH for F1-ORF13 was determined using APTS-labelled FA2G0-SO_4_, following product formation by xCGE-LIF. **C** F1-ORF13 ion requirement. Activity of F1-ORF13 was assayed in the presence of different metal ions. Formation of the reaction product was monitored by xCGE-LIF. The x-axis (time) of the electropherograms was normalized to two internal standards by glyXtool^CE^, resulting in double normalized migration time units (MTU”). Signal Intensities were normalized to the total peak height, resulting in relative peak height proportions (%). Peaks marked with (*) correspond to the internal standard used for migration time normalization in xCGE-LIF. Glycans are represented using the SNFG nomenclature [69]

While our screen was designed to isolate enzymes that act upon a sulfated monosaccharide analog 4MU-GlcNAc-6-SO_4_, a broader aim of the study was to find enzymes that act on sulfated GlcNAc in the context of an intact *N*-glycan. As such, the activity of the F1-ORF13 sulfatase was evaluated on a sulfated *N*-glycan substrate isolated from a mammalian glycoprotein via analysis by electrophoretic separation using xCGE-LIF. To enable this experiment, the F1-ORF13 sulfatase was cloned and expressed in vivo in *E. coli* (Additional file 1: Fig. S6). Partially purified enzyme was incubated with *N*-glycans enzymatically released from human immunoglobin A (hIgA) and reductively labelled with the fluorophore aminopyrene trisulfonic acid (APTS). HIgA contains 2 or 5 *N*-glycans per heavy chain for the subclasses A1 or A2, respectively [4]. The pool of glycans released from hIgA contains a form of a sialylated di-antennary *N*-glycan having a single sulfate group on one outer-arm GlcNAc residue (referred to as FA2G2S2-SO_4_) (Cajic S, Hennig R, Grote V, Reichl U and Rapp E; manuscript in preparation). To create a substrate with sulfated GlcNAc positioned in the terminal position of the outer arm, FA2G2S2-SO_4_ was sequentially treated with sialidase A and β(1–4,6)-galactosidase to generate FA2G2-SO_4_ and FA2G0-SO_4_ substrates, respectively. Enzymatically treated and untreated *N*-glycans were analyzed and compared using glyXbox^CE^, based on xCGE-LIF. The F1-ORF13 sulfatase cleaved sulfate from terminal GlcNAc on FA2G0-SO_4_ (Fig. 6A top panel) in a concentration-dependent manner (Additional file 1: Fig. S7) but did not show activity on FA2G2-SO_4_ (Fig. 6A middle panel) or FA2G2S2-SO_4_ (Fig. 6A bottom panel). F1-ORF 13 was also unable to remove sulfate from an APTS-labelled *N*-glycan released from human urokinase (Additional file 1: Fig. S8A). In this protein the dominant *N*-glycan [termed FA2FGalNAc-(SO_4_)_2_] contains two sulfated terminal GalNAc residues, with an additional antenna fucose at the adjacent GlcNAc [47, 48]. Finally, F1-ORF13 was tested on a mixture of total *N*-glycans released from hIgA and subsequently desialylated and degalactosylated. In this context, F1-ORF13 was also able to hydrolyze SO_4_ from terminal GlcNAc-SO_4_ (Fig. S9). These experiments support the conclusion that the F1-ORF13 sulfatase can remove sulfate from GlcNAc-6-SO_4_ in the context of an intact *N*-glycan, but only if it is the terminal residue in an outer-arm branch.

The FA2G0-SO_4_ substrate was also used to determine pH and ion requirements of F1-ORF13 via xCGE-LIF. Reactions were performed in triplicate at each pH and yield was measured by integration of the peaks observed by xCGE-LIF (Additional file 1: Fig. S10). The enzyme is active over pH 5–10 with a peak around pH 8–9 (Fig. 6B). Consistent with other proteins of this enzyme family, F1-ORF13 is calcium-dependent (Fig. 6C) [49, 50]. No hydrolysis of sulfate from FA2G0-SO_4_ was detected unless Ca^2+^ ions were present (Fig. 6C). Nevertheless, in absence of calcium, the height of the peak corresponding to FA2G0-SO_4_ decreased upon addition of F1-ORF13 sulfatase (Additional file 1: Fig. S11A) suggesting that F1-ORF13 was binding to the substrate.

### F1-ORF13 binds GlcNAc-6-SO_4_-containing *N*-glycans in absence of calcium

While testing F1-ORF13 metal ion requirement via xCGE-LIF, F1-ORF13 was incubated with APTS-labelled FA2G0-SO_4_ *N*-glycans in absence of calcium to serve as a control. During this experiment we noticed that the peak corresponding to FA2G0-SO_4_ was decreasing but not shifting in migrating time. We hypothesized that in the absence of Ca^2+^, F1-ORF13 bound sulfated-GlcNAc *N*-glycans. The complex formed would be too large to be eluted during the HILIC-SPE clean-up performed before each xCGE-LIF run. To test this hypothesis, different amounts of F1-ORF13 were incubated with FA2G0-SO_4_ in the absence of calcium. After incubation, samples were either directly cleaned-up with HILIC-SPE or first digested with proteinase K and then, cleaned with HILIC-SPE (Additional file 1: Fig. S11). Proteinase K digestion was used to destroy F1-ORF13 and thus disrupt a possible F1-ORF13-FA2G0-SO_4_ complex.

Samples directly cleaned with HILIC-SPE, showed a clear decrease in the amount of FA2G0-SO_4_ (Additional file 1: Fig. S11A). Reduction of the amount of substrate was F1-ORF13 concentration-dependent. In the samples treated with proteinase K followed by HILIC-SPE clean-up, no significant change in the relative substrate peak height was detected (Additional file 1: Fig. S11B). This confirmed the inability of F1-ORF13 to hydrolyze SO_4_ from GlcNAc in the absence of calcium as well as its capacity to recognize and bind to GlcNAc-SO_4_-containing glycans. This characteristic of F1-ORF13 expands its potential as a tool to study *N*-glycan sulfation. For example, in the absence of calcium it can be used as a terminal GlcNAc-SO_4_ *N*-glycan-binding protein while in the presence of calcium it is a GlcNAc-SO_4_ sulfatase. The use of an enzyme in its apo form is an approach already employed for some nonconsuming substrate sensors [51].

### Identifying genes encoding active hexosaminidases using in vitro protein expression

**Fig. 7:**
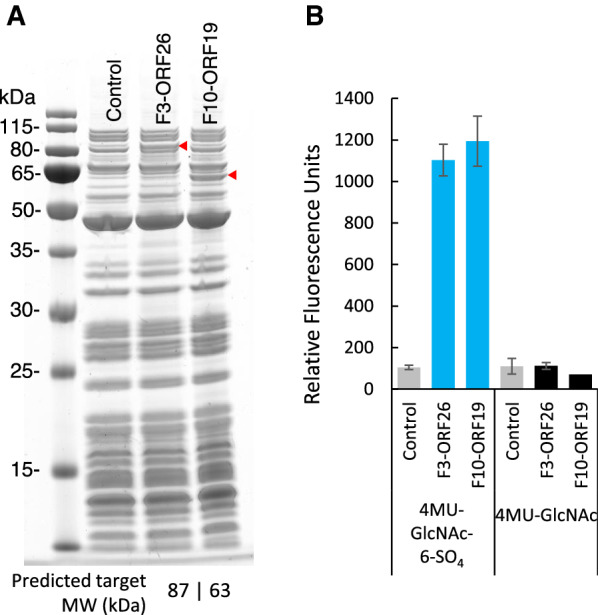
In vitro expression of hexosaminidase clones F3-ORF26 and F10-ORF19. **A** SDS-PAGE of 2 hexosaminidases produced with the PURExpress® system. Expressed enzymes are shown with a red triangle. **B** Activity of in vitro expressed hexosaminidases. Hexosaminidases were assayed on 4MU-GlcNAc-6-SO_4_ and its asulfated counterpart 4MU-GlcNAc

Two hexosaminidase candidates were investigated: F3-ORF26 (a gene common to F3, F4 and F9, described above) and F10-ORF19. Neither of the two putative sulfatases encoded by clone F10 showed statistically significant activity on 4MU-GlcNAc-6-SO_4_ (Fig. 4B). Yet, clone F10 demonstrated activity on 4MU-GlcNAc-6-SO_4_ in the presence and in the absence of exogenous hexosaminidase (Fig. 3A). We hypothesized that the putative hexosaminidase gene also encoded by this clone (F10-ORF19) might be solely responsible for the fluorescent signal detected during screening. To test candidate hexosaminidase genes for activity, both genes (F3-ORF26 and F10-ORF19) were expressed in vitro using the PURExpress® system and the produced proteins were assayed (Fig. 7).

Both F3-ORF26 and F10-ORF19 were produced efficiently in the PURExpress® system (Fig. 7A). Furthermore, they both hydrolyzed 4MU-GlcNAc-6-SO_4_ indicating they are not inhibited by the presence of C6 sulfate on GlcNAc (Fig. 7B). Interestingly, both enzymes showed no activity on asulfated 4MU-GlcNAc, indicating they likely require sulfated GlcNAc for hydrolysis.

### Protein sequence analysis of active hexosaminidases

The protein sequences of F10-ORF19 and F3-ORF26 hexosaminidases were compared and were 62.5% similar (Additional file 1: Fig. S12A). F3-ORF26 contained a C-terminal region of ~ 230 amino acids absent in F10-ORF19. Both proteins belong to glycoside hydrolase family 20 (GH20) and perfectly matched annotated N-acetyl-hexosaminidases protein sequences from *Bacteroides caccae* (F10-ORF19) and *Phocaeicola dorei* (F3-ORF26). To the best of our knowledge, neither the *Bacteroides caccae* nor *Phocaeicola dorei* hexosaminidases have been previously biochemically characterized. However, hexosaminidases from other organisms that are able to hydrolyze sulfated GlcNAc residues have been reported under the names ‘sulfoglycosidase’ or ‘mucin-desulfating glycosidase’ [52, 53]. Originating from *Prevotella* or *Bifidobacterium bifidum* these enzymes were tested on synthetic p-nitrophenyl substrates or mucin type *O*-glycans but not on more complex structures like sulfated *N*-glycans. A phylogenetic tree showing the evolutionary relationship between F10-ORF19, F3-ORF26 and other GH20 members is shown in Additional file 1: Fig. S12B. The F10-ORF19 hexosaminidase from *Bacteroides caccae* was selected for further biochemical characterization on sulfated *N*-glycans.

### F10-ORF19 hexosaminidase activity upon GlcNAc-6-SO_4_ in intact *N*-glycans

**Fig. 8:**
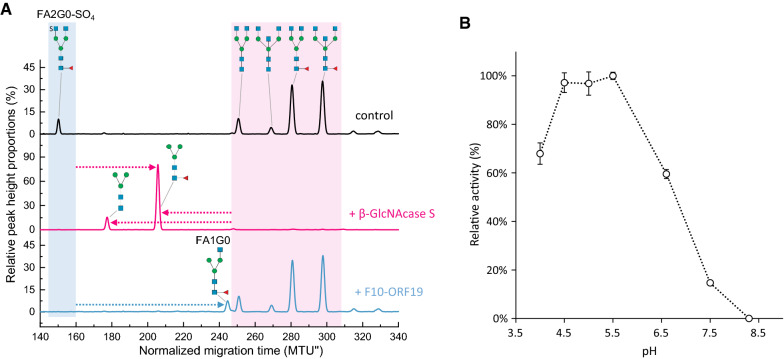
F10-ORF19 hexosaminidase activity on *N*-glycans. **A** F10-ORF19 hexosaminidase substrate specificity. F10-ORF19 was assayed on a pool of human APTS-labelled immunoglobulin A (hIgA) *N-*glycans pretreated with sialidase and β-galactosidase (black electropherogram). Substrate and product were analyzed by xCGE-LIF. The activity of F10-ORF19 (blue electropherogram) is indicated with a blue arrow that shows the migration time shift of the FA2G0-SO_4_ peak from ~ 150 MTU″ to the FA1G0 peak at ~ 245 MTU″ due to the loss of a sulfated GlcNAc. The activity of β-N-acetylhexosaminidase S (β-GlcNAcase S; pink electropherogram) is shown with 3 pink arrows illustrating the collapse of all structures to paucimannose at (~ 178 MTU″) and fucosylated paucimannose (~ 207 MTU″), respectively. Structure assignment was enabled by matching the normalized migration times with those of a *N*-glycan database and confirmed by exoglycosidase digests (Cajic S, Hennig R, Grote V, Reichl U and Rapp E; manuscript in preparation). **B** F10-ORF19 optimal pH. Optimal pH for F10-ORF19 was determined using the hIgA-APTS labelled glycan pool as substrate and following product formation by xCGE-LIF. The x-axis (time) of the electropherograms was normalized to two internal standards by glyXtool^CE^, resulting in double normalized migration time units (MTU″). Signal Intensities were normalized to the total peak height, resulting in relative peak height proportions (%). Glycans are represented using SNFG nomenclature [69]

F10-ORF19 hexosaminidase was expressed in vivo in *E. coli* and purified to be tested in the context of *N*-glycans. The activity of F10-ORF19 hexosaminidase was investigated by xCGE-LIF on the pool of hIgA APTS-labelled glycans previously digested with both sialidase A and β(1–4,6)-galactosidase to expose GlcNAc-SO_4_ at the terminal end (Fig. 8A black electropherogram). F10-ORF19 was capable of hydrolyzing GlcNAc-SO_4_ while showing no activity on asulfated GlcNAc (Fig. 8A blue electropherogram). In the presence of a large excess of enzyme, trace activity on asulfated GlcNAc is observed (data not shown) but in the conditions reported here, the enzyme showed selectivity for sulfated GlcNAc residues. This specificity contrasts with that of a well-characterized hexosaminidase from *Streptococcus pneumoniae* that is commonly used in glycoanalytics (β-*N*-acetylglucosaminidase S) and hydrolyzed both sulfated and non-sulfated GlcNAc as well as bisecting GlcNAc (Fig. 8A shaded pink box). F10-ORF19 was active from pH 4 to 7 with an optimal activity around pH 5 (Fig. 8B). F10-ORF19 also showed no activity on urokinase APTS-labelled FA2FGalNAc-(SO_4_)_2_ (Additional file 1: Fig. S8B) indicating it does not act upon sulfated GalNAc and is specific for GlcNAc-6-SO_4_.

## Discussion

We created a human enteric metagenomic library and explored it for clones encoding sugar-specific sulfatases using function-based enzyme screening with a coupled assay strategy. We sought enzymes that could address site-specific sulfation in *N*-glycans and potentially be used as analytical tools. Our screen identified both sulfatases and hexosaminidases that act specifically upon GlcNAc-6-SO_4_. Two enzymes isolated from our screen, a sulfatase (F1-ORF13) and a hexosaminidase (F10-ORF19), were cloned, purified, and further characterized. We showed that F1-ORF13 is a calcium-dependent sulfatase that exclusively removes sulfate from carbon-6 of GlcNAc. The enzyme will catalyze sulfate removal from both a GlcNAc-6-SO_4_ monosaccharide or GlcNAc-6-SO_4_ when it is in the terminal position of an *N*-glycan outer-arm. Furthermore, in the absence of Ca^2+^ ions, the apo-enzyme will act as a GlcNAc-6-SO_4_ glycan binding protein (GBP). Lectins and GBPs have been employed in glycoanalytic workflows to enrich or deplete glycans/glycopeptides bearing specific epitopes [27, 29, 54] from a complex mixture but none specifically address sulfated glycosylation to date. This feature is of particular interest considering the low abundance of sulfated glycans in most biological samples. Traces of GlcNAc-6-SO_4_-containing *N*-glycans could be captured prior to analysis, enabling their detection otherwise concealed by non-sulfated structures. An additional study further evaluating F1-ORF13 as a GlcNAc-6-SO_4_ GBP for use in glycoanalytics is ongoing. Finally, we demonstrated that the F10-ORF19 hexosaminidase is capable of hydrolyzing GlcNAc-6-SO_4_ from the terminal position of *N*-glycans while showing negligible activity on asulfated GlcNAc. Considered together, the narrow specificity of both enzymes for terminal GlcNAc-6-SO_4_ in *N*-glycans and their ability to be used with other exoglycosidases highlights their utility as novel tools to confirm the presence or absence of GlcNAc-6-SO_4_ in glycoanalytics.

The F1-ORF13 sulfatase belongs to the S1 family of sulfatases in which members possess an intriguing modification of a critical serine or cysteine to formylglycine (FGly) forming two subtypes Ser-type and Cys-type S1 sulfatases [49, 55]. Discovered by von Figura’s group in 1995, the FGly modification is unique to S1 sulfatases and thought to be essential for their activity [56]. To date, two main enzymatic systems that enable formation of FGly have been identified: (1) formylglycine generating enzymes (FGEs) found in eukaryotes and some prokaryotes, and (2) anaerobic sulfatase maturating enzymes (anSMEs) exclusively found in prokaryotes [57, 58]. Surprisingly, in our work, expression of active F1-ORF13 was obtained both in vitro and in vivo in *E. coli* in the absence of an obvious FGE or anSME. Two hypotheses may explain these observations. First, *E. coli* might have an intrinsic ability to convert F1-ORF13 Ser94 to FGly. Similar observations have been reported for an active Klebsiella Ser-type sulfatase that was successfully produced in *E. coli* [59]. Sulfatase maturation systems in *E. coli* are still not completely understood, yet two putative anSME proteins (aslB and ydeM) have been described, and a third, still unidentified FGly-forming system is suspected to exist [57]. We also investigated the potential presence of a putative sulfatase maturation system within fosmid F1. While none of the 29 ORFs predicted on F1 shared homology with known FGEs or anSMEs, F1-ORF2 encoded a hypothetical protein containing a predicted 4Fe-4S cluster, a known hallmark of sulfatase maturating enzymes [60]. However, our initial attempts to co-express F1-ORF2 and F1-ORF13 did not increase the activity of F1-ORF13 (data not shown). A second hypothesis is that the F1-ORF13 sulfatase might possess some activity even in the absence of the FGly modification. This notion is supported by the activity of F1-ORF13 we detect after in vitro expression.

The assay system we used in this study proved successful at identifying enzymes that act on or require a highly specific chemical modification of GlcNAc in high-throughput screening. While functional metagenomics identified enzyme candidates, xCGE-LIF was used to evaluate their capability to act on real *N*-glycan substrates. Extending the activity testing of enzyme candidates to natural carbohydrates was essential to prove their utility in glycoanalytics. Our screening assay permitted identification of enzymes that address sulfation of the 6-carbon of GlcNAc. However, it could be easily adapted to screen for enzymes that address different chemical modifications, a different position of modification, or modification of other sugars. For example, it would be of interest to identify sulfatases that can specifically act on GalNAc-4-SO_4_ or Gal-3-SO_4_, both modifications typically found on *N*-glycans, a class of glycoconjugates for which the impact of sulfate groups has been poorly studied [14]. In addition, the assay could be extended to identify enzymes that act upon sugars having other PGMs (e.g., methyl, acetyl, phosphate, etc.) that are found in a wide range of eukaryotic glycans [61]. Furthermore, little is known about enzymes that hydrolyze zwitterionic glycan modifications (e.g., phosphoethanolamine, phosphocholine) that are commonly found on glycolipid anchor glycans [62], bacterial biofilm cellulose [63] and invertebrate *N*-glycans [61]. The biological importance of PGM modifications appears vast [13, 64] and expansion of the analytical enzyme toolbox to enable their characterization will help the field gain further insight into the roles of PGMs in glycobiology.

## Conclusions

We used functional metagenomics screening to identify enzymes that act upon GlcNAc-6-SO_4_, a glycan modification observed in both *N*- and *O*-glycans. Using glycoanalytics, we showed that two identified enzymes, a sulfatase (F1-ORF13) and a hexosaminidase (F10-ORF19), were able to remove sulfate from carbon-6 of GlcNAc and GlcNAc-6-SO_4_ from the terminal position of *N*-glycans, respectively. Additionally, the F1-ORF13 sulfatase binds to GlcNAc-6-SO_4_ in absence of its calcium cofactor, a promising feature for the enrichment of GlcNAc-6-SO_4_-containing glycans or glycopeptides. The precise specificities of these enzymes and their ability to act on GlcNAc-6-SO_4_ in *N*-glycans highlights their potential as tools to enhance *N*-glycan structural analysis.

## Methods

*Chemicals, reagents and enzymes* Lysozyme, phenol:chloroform:isoamyl alcohol, 8-aminopyrene-1,3,6-trisulfonic acid (APTS), IGEPAL^®^ CA-630, triethylamine (TEA), acetic acid and isopropyl β-D-1-thiogalactopyranoside (IPTG) were from MilliporeSigma. RNase A was from Qiagen (Venlo, Netherlands). PNGaseF, proteinase K, β-agarase, FseI, β-N-Acetylhexosaminidase_f_, Monarch® PCR and DNA clean-up kit, PURExpress® in vitro protein synthesis kit, RNase inhibitor murine, NEBuilder® HiFi DNA assembly cloning kit, GlycoBuffer 1, Q5® Hot Start High-Fidelity 2X Master Mix, NEB 5 alpha competent cells and NEBExpress® Ni spin columns were from New England Biolabs (Ipswich, MA, USA). 4-Methylumbelliferyl N-acetyl-β-D-glucosaminide-6-sulfate (4MU-GlcNAc-6-SO_4_), 4-Methylumbelliferyl N-acetyl-β-d-glucosaminide (4MU-GlcNAc), d-glucosamine-6-O-sulfate (GlcN-6-SO_4_), N-acetyl-d-galactosamine-4-O-sulfate (GalNAc-4-SO_4_) and d-galactose-4-O-sulfate (Gal-4-SO_4_) substrates were from Dextra Laboratories Ltd (Reading, UK). N-acetyl-d-glucosaminide-6-O-sulfate (GlcNAc-6-SO_4_) was from Sigma. AMPure® PB magnetic beads, Pacific Bioscience barcoded adapter and all reagents for MagBead complex preparation were from Pacific Bioscience (Menlo Park, CA, USA). CopyControl™ Fosmid Library Production kit and inducing solution were from Lucigen Corporation (Middleton, WI, USA). Immunoglobulin A from human plasma (hIgA) was from Athens Research and Technology (Athens, GA, USA). Human urokinase was from Active Bioscience (Hamburg, Germany). Reducing agent: ReduX Solution, HILIC-SPE material: glyXbeads, and xCGE-LIF migration time normalization standard: 2nd NormMiX were from glyXera GmbH (Magdeburg, Germany). Sialidase A and β(1–4,6)-galactosidase were from Agilent (Santa Clara, CA, USA). Phosphate-Buffered Saline (PBS), Y-PER™ yeast protein extraction reagent, GeneScan™ 500 LIZ™ size standard, Hi-Di™ formamide and POP-7™ polymer and Slide-A-Lyzer dialysis cassettes were from ThermoFisher Scientific. HisTrap Fast Flow columns were from Cytiva (Malborough, MA, USA). Milli-Q (MQ) water was from (Merck Millipore, Germany).

### Human gut microbiome DNA isolation

Human microbiome DNA was isolated from a fecal sample (Donor T3806, Lee Biosolutions, Maryland Heights, MO, USA) of a healthy 29-year-old man. Microbial cells were extracted from 100 mg of fecal material using PBS as detailed in the supplementary information. To proceed with cell lysis, the cell pellet was resuspended in 500 µL of light TE buffer (10 mM Tris–HCl pH 8.0, 0.1 mM EDTA). To this cell suspension, 100 µL of lysozyme (10 mg/mL) and 1 µL of RNAse A (100 mg/mL) were added. The sample was incubated at 37 °C for 30 min, after which, 20% SDS was added to a 0.5% final concentration. Proteinase K in a ratio of 40 µL per milliliter of sample was added and incubated for 20 min at 37 °C. Lysate was centrifuged at 10,000×*g* for 10 min at 4 °C to pellet cell debris. The supernatant was transferred to a clean tube with a wide bore pipette tip to avoid shearing the DNA. DNA was isolated by addition of an equal volume of phenol:chloroform:IAA (25:24:1). For cleaner DNA, phenol:chloroform:IAA extraction was performed twice followed by isopropanol precipitation. DNA of 30–70 kb was size selected from a 1% low melting point agarose gel run overnight at 4 °C at 35 V. DNA was recovered from the gel using 1 U of β-agarase for each 100 µL of melted agarose.

### Metagenomic library construction

The metagenomic library was constructed using the CopyControl™ Fosmid Library Production kit as recommended and described [65]. Generated clones were then packaged into λ phages for transfection of *E. coli* following the manufacturer’s instructions. A small portion of the packaging reaction was used to transfect EPI300-T1^R^ cells to determine the phage titer. The remainder was stored as a 20% glycerol stock at − 80 °C until large scale transfection of EPI300-T1^R^ was performed. From the large-scale transfection, 23,040 clones were picked and archived in 60 × 384 well plates as micro-culture glycerol stocks by BioS&T (Montreal, Canada).

To assess the quality of the library, 24 clones were picked and their fosmids isolated using the FosmidMAX DNA purification kit. One microgram of fosmid DNA was digested with 2U of FseI restriction enzyme and then separated on a 1% agarose gel. For Sanger sequencing 100 ng of fosmid DNA were mixed with the T7 universal primer or the pCC1 reverse primer (Lucigen corporation) (Additional file 1: Table S1) in 10 µL reactions.

### Screening for sulfated glycan using a coupled assay

Clones from the metagenomic library were cultured in 384 well plates with 50 µL LB (10 g tryptone, 5 g yeast extract, 10 g NaCl, 1 g dextrose, 1 g MgCl_2_-6H_2_O, 2 mL of 2 M NaOH per liter) containing 12.5 μg/mL chloramphenicol and 1X inducing solution. After overnight incubation at 37 °C, 50 µL of Y-PER™ lysis buffer supplemented with 40 μg/mL of 4MU-GlcNAc-6-SO_4_ and 1 U/mL of β-*N-*Acetylhexosaminidase_f_ were added to each well. The 384 well plates were incubated at 37 °C in a static incubator for 48 h. The fluorescence signal emitted from each plate was monitored about six times (typically after 1, 3, 6, 24, 30 and 48 h) over the course of the experiment using a SpectraMax Plus 384 Microplate reader (Molecular Devices, San Rose, CA, USA).

### PacBio sequencing

Fosmid DNA was isolated using the FosmidMAX DNA purification kit following the manufacturer’s instructions. Two 10-kb multiplexed SMRT-bell libraries were prepared, each enabling sequencing of 12 fosmids using a single SMRT cell. Fosmid DNA was first sheared into 10-kb pieces using g-tubes (Covaris, Woburn, MA, USA) as described [54]. Multiplexed libraries were prepared as recommended by PacBio. Briefly, each fosmid was individually end-repaired and ligated to PacBio barcoded adapters. After ligation, all twelve reactions were pooled together. Damaged DNA was repaired and fragments not ligated to SMRT bell adaptors degraded using exonucleases. To remove SMRT-bell templates smaller than 8 kb from the SMRT-bell libraries, a BluePippin size selection (Sage Science, MA, USA) was performed following the manufacturer’s instructions using 0.75% agarose cassette.

Size-selected libraries were sequenced on a Pacific Bioscience RSII instrument with the P6 chemistry. One single-molecule real-time (SMRT) cell was used for each library and data was collected for 360 min. For each library, reads were first demultiplexed into 12 sets of reads, one set per barcode, each containing data for a single fosmid. After demultiplexing, the HGAP.3 protocol was used to de novo assemble the reads. Contigs were circularized with the Circlator program [66]. Finally, ORFs were predicted using MetaGeneMark [67]. Annotation of predicted ORFs was performed by BLASTp against the entire NCBI protein database; ORFs with a significant match to proteins with known function were annotated with the same function; ORFs with no significant match to known proteins were annotated as hypothetical. An ORF map was thus created for each properly assembled fosmid using the Geneious software (https://www.geneious.com) after the sequence of the pCC1 backbone was trimmed.

### In vitro expression of ORFs of interest

ORFs of interest were expressed in vitro using the PURExpress® kit. ORFs were amplified from the fosmid using primers designed following the manufacturer’s instructions (Additional file 1: Table S1). After PCR, linear templates were purified with the Monarch PCR & DNA clean-up kit. A 50 µL PURExpress® reaction was then prepared by mixing 500 ng of template, 20 µL of solution A, 15 µL of solution B and 1 µL of RNase inhibitor murine. In vitro expression was performed for 2 h at 37 °C. In vitro expressed proteins were then assayed in triplicate using 10 µL of PURExpress® mixture, 2 µL of 4MU-GlcNAc-6-SO_4_ or 4MU-GlcNAc (100 µg/mL) for hexosaminidase activity. For sulfatase activity 5U of β-N-Acetylhexosaminidase_f_ were also added to the reactions. Incubation was at 37 °C and activity determined by measuring fluorescence at 365/445 nm using a SpectraMax Plus 384 microplate reader (Molecular Devices, San Rose, CA, USA).

### F1-ORF13 sulfatase and F10-ORF19 hexosaminidase in vivo expression and purification

F1-ORF13 sulfatase and F10-ORF19 hexosaminidase were cloned for in vivo expression. F1-ORF13 was synthesized with codon optimization for expression in *E. coli* and cloned with a C-terminal 6xHis-tag into a pET21a( +) vector by GenScript (Piscataway, NJ, USA). F10-ORF19 hexosaminidase was cloned without its predicted signal sequence (residues 1–22) and with a C-terminal 6-His-tag using the NEBuilder^®^ HiFi DNA Assembly Cloning kit. Primers were designed using the NEBuilder^®^ online tool (https://nebuilder.neb.com/) (Additional file 1: Table S1). The pET28c( +) vector was modified to contain a transcription terminator sequence upstream of the promoter region for tighter regulation of protein expression.

F1-ORF13 and F10-ORF19 were expressed in NEB T7 Express *E. coli* cells. For each ORF, a 1L culture of LB containing 100 µg/mL ampicillin was inoculated with a single colony and induced for protein expression by addition of IPTG to 0.4 mM final concentration once the culture OD_600_ reached 0.4–0.6. Induction was performed overnight at 16 °C. Cells were harvested and resuspended in loading buffer (20 mM sodium phosphate, 500 mM NaCl, 20 mM imidazole, pH 7.4). Lysis was performed using a HPL6 High Pressure Homogenizer (Maximator GmbH, Nordhausen, Germany) for 3 passes at 10 kPsi. Proteins were purified on a HisTrap Fast Flow column. Bound proteins were eluted from the column with a linear gradient of imidazole concentrations from 20 to 500 mM. Fractions containing the protein of interest were pooled and dialyzed against imidazole-free buffer (20 mM sodium phosphate, 500 mM NaCl, 1 mM EDTA, pH 7.4) using a 3.5 kDa MWCO Slide-A-Lyzer dialysis cassette.

### F1-ORF13 activity on sulfated monosaccharides

Specificity of F1-ORF13 was tested on the following sulfated monosaccharides: GlcNAc-6-SO_4_, GlcN-6-SO_4_, GalNAc-4-SO_4_, GalNAc-6-SO_4_ and Gal-4-SO_4_. Reactions were performed in 20 µL volume with 10 µL of in vitro PURExpressed F1-ORF13 or PURExpress® control (no DNA template) mixtures in 25 mM HEPES pH 8.3, 10 mM CaCl_2_ with 160 µM sulfated monosaccharide. Incubation was at 37 °C overnight. Proteins were precipitated by addition of 80 µL of cold acetone to each 20 µL reaction and incubation at − 20 °C for 1 h. Samples were centrifuged at 14,000×*g* for 10 min at 4 °C and supernatant recovered. An additional 500 µL of acetone was added to the pellet, samples were quickly vortexed and centrifuged a second time. The supernatant was recovered and pooled with the previous one. Pooled supernatants were dried in a speed vacuum concentrator (Martin Christ Gefriertrocknungsanlagen GmbH, Osterode am Harz, Germany) and resuspended in 125 µL of water. Samples were analyzed on a Dionex ISC-5000 + a high-performance anion exchange chromatography system with pulsed amperometric detection (HPAEC-PAD) (see Additional file 1 for further details).

### Enzyme activities on *N*-glycans

#### *N*-glycan release

*N*-glycans were released from hIgA or human urokinase with PNGase F. To that end, four aliquots each containing 420 µg of urokinase or 210 µg of hIgA were mixed with 2% SDS for a final SDS concentration of ~ 0.3%. Aliquots were incubated for 10 min at 60 °C. SDS was then neutralized by addition of 8% IGEPAL^®^ in PBS to a final concentration of 1.8%. Enzymatic glycan release was achieved with 7 U of PNGase F and aliquots incubated for 3 h at 37 °C.

#### APTS-labelling

Released *N*-glycans were dried and labelled via reductive amination with 1-aminopyrene-3,6,8-trisulfonic acid APTS as previously described [68]. Briefly, 28 µL of MQ water, 28 µL of 20 mM APTS in 3.6 M citric acid and 28 µL of reducing agent were added to each of the four aliquots. Labelling was performed for 3 h at 37 °C. A volume of 1.4 mL of 80% acetonitrile (in water) was added to each of the four aliquots prior to pooling them together. Labelled *N*-glycans were cleaned-up using HILIC-SPE as previously described [68].

#### *N*-glycan fractionation

Labelled glycans were fractionated on a Dionex Ultimate 3000 HPLC equipped with a Jasco FP-2020 Plus fluorescence detector (see details in Additional file 1). Fractions containing *N*-glycans of interest were dried and resuspended with MQ water for use as enzyme substrates.

#### Substrate preparation

Substrates used for testing F1-ORF13 activity were the following APTS-labelled *N*-glycans: desialylated and degalactosylated APTS-labelled hIgA *N*-glycan pool, FA2G2S2-SO_4_, FA2G2-SO_4_, FA2G0-SO_4_ and an *N*-glycan composed of the core FA2 with one branched fucose and two terminal sulfated GalNAcs (termed here FA2FGalNAc-(SO_4_)_2_). FA2G2S2-SO_4_ and FA2FGalNAc-(SO_4_)_2_ were isolated from hIgA and urokinase respectively by *N*-glycan fractionation as described in the supplementary information. FA2G2-SO_4_ was prepared from FA2G2S2-SO_4_ by digestion with sialidase A. Purified FA2G2S2-SO_4_ resulting from fractionation was dried, treated with sialidase A as recommended by the supplier and digest performed overnight at 37 °C. Digested *N*-glycans were cleaned-up by HILIC-SPE as described previously [68] with the exception that the 5 washes were omitted. FA2G0-SO_4_ was subsequently prepared by digesting FA2G2-SO_4_ with β(1–4,6)-galactosidase, following the supplier instructions. Reaction was incubated overnight at 37 °C and cleaned-up by HILIC-SPE as described for the sialidase A digest. Total *N*-glycans from hIgA were desialylated and degalactosylated using sialidase A and β(1–4,6)-galactosidase as described above.

Substrates for F10-ORF19 testing were desialylated and degalactosylated APTS-labelled total *N*-glycans released from hIgA and urokinase FA2FGalNAc-(SO_4_)_2_ APTS-labelled glycan obtained by fractionation.

#### *F1-ORF13 sulfatase activity on N*-glycans released from hIgA and human urokinase

F1-ORF13 was assayed separately using APTS-labelled desialylated and degalactosylated hIgA *N*-glycan pool, FA2G2S2-SO_4_, FA2G2-SO_4_, FA2G0-SO_4_ from hIgA and FA2FGalNAc-(SO_4_)_2_ from human urokinase. Each 10 µL reaction contained 4 µL of substrate, 4 µL of 50 mM HEPES buffer, pH 8.3, 1 µL of 100 mM CaCl_2_ and 6 µg of F1-ORF13. Reactions were incubated at 37 °C for 3 h and cleaned-up with HILIC-SPE. Samples were dried in a speed vacuum concentrator and resuspended with 15 µL of MQ water prior to xCGE-LIF.

F1-ORF13 optimal pH was determined using the APTS-labelled FA2G0-SO_4_ *N*-glycan in 10 µL reactions containing 4 µL of 50 mM buffer, 1 µL of 100 mM CaCl_2_ and 6 µg of F1-ORF13. Buffers tested were sodium acetate, pH 4.0 and 5.0, MES buffer, pH 5.5 and 6.6, sodium phosphate buffer, pH 7.5, HEPES, pH 8.3 and Tris buffer, pH 9.0. After 3 h incubation at 37 °C, reactions were cleaned-up by HILIC-SPE and product formation monitored by xCGE-LIF.

To test F1-ORF13’s metal ion requirement, 10 µl reactions were performed as described above using HEPES pH 8.3 as buffer and 1 µL of 100 mM solution of CaCl_2_, MgCl_2_, MnSO_4_ or ZnSO_4_. 1 µL of MQ water was used for the control sample. Incubation was performed for 3 h at 37 °C prior to HILIC-SPE clean-up and xCGE-LIF analysis.

To investigate the ability of F1-ORF13 to bind the APTS-labelled FA2G0-SO_4_ *N*-glycan substrate in absence of calcium, 10 µL solutions containing 2 µL of MES buffer pH 5.5 and 0.6 or 6 µg F1-ORF13 were incubated at 37 °C for 3 h. Reactions were performed in duplicate. One set of reactions was directly subjected to HILIC-SPE, the other was digested with 1 µL of 20 mg/mL proteinase K (AppliChem GmbH, Darmstadt, Germany). Proteinase K treated samples were incubated for 2 h at 42 °C, cleaned with HILIC-SPE, and analyzed by xCGE-LIF.

#### *F10-ORF19 activity on N*-glycans released from hIgA and human urokinase

F10-ORF19 was assayed on the desialylated and degalactosylated APTS-labelled hIgA *N*-glycan pool or APTS-labelled urokinase FA2FGalNAc-(SO_4_)_2_ N-glycan in 10 µL reactions. Reactions were performed in GlycoBuffer 1 using 1 µg of F10-ORF19. Incubation was performed at 37 °C for 1 h. Prior to xCGE-LIF measurement, samples were cleaned-up with HILIC-SPE as described previously [68].

F10-ORF19 optimal pH was determined using the desialylated and degalactosylated APTS-labelled hIgA *N*-glycan pool as the substrate. 10 µL reactions with 4 µL of 50 mM buffer and 0.1 µg of F10-ORF19 were incubated for 30 min at 37 °C. Tested buffers were: sodium acetate, pH 4.0, 4.5 and 5.0, MES buffer, pH 5.5 and 6.6, sodium phosphate, pH 7.5 and HEPES buffer, pH 8.3. Prior to xCGE-LIF analysis, samples were cleaned-up with HILIC-SPE as described previously [68].

#### xCGE-LIF analysis

xCGE-LIF-based glycoanalysis was performed as described [68]. Samples were run on glyXbox^CE^ systems (glyXera GmbH, Magdeburg, Germany) based on modified Applied Biosystems 3130 or 3130xl genetic analyzers using a 50 cm capillary array filled with POP-7™ polymer. For injection, samples were prepared using 1–3 µL of HILIC-SPE purified APTS-labelled samples, adding 0.5 µL of GeneScan™ 500 LIZ™ size standard diluted 1:50 in Hi-Di™ formamide, 0.5 µL of xCGE-LIF migration time normalization standard 2nd NormMiX and Hi-Di™ formamide up to a total sample volume of 12 µL. Samples were electrokinetically injected and separation was at 30 °C at 15 kV. Data were analyzed using the glycoanalysis software glyXtool^CE^ (glyXera).

## Supplementary Information

**Additional file 1.** Additional methods table and figures.

## Data Availability

The sequencing data supporting the conclusions of this article are available in the GenBank™/EBI Data Bank repository, [MW677166-MW677184 in https://www.ncbi.nlm.nih.gov/genbank/].
