# Supplementary material for: Combining functional metagenomics and glycoanalytics to identify enzymes that facilitate structural characterization of sulfated N-glycans
Source: Microb Cell Fact. 2021 Aug 21;20:162. doi: 10.1186/s12934-021-01652-w (PMC8379841; doi:10.1186/s12934-021-01652-w)
Supplement: Supplementary file 1 — Additional file 1. Additional methods table and figures. [file 12934_2021_1652_MOESM1_ESM.pdf]

Supplementary information

Combining functional metagenomics and glycoanalytics to identify enzymes that facilitate structural characterization of sulfated *N*-glycans

Léa Chuzel[1,2], Samantha L. Fossa[2], Madison L. Boisvert[2], Samanta Cajic[1], René Hennig[3], Mehul B. Ganatra[2], Udo Reichl[1,4], Erdmann Rapp[1,3], Christopher H. Taron[2*]

[1]Bioprocess Engineering, Max Planck Institute for Dynamics of Complex Technical Systems, 39106 Magdeburg, Germany

[2]New England Biolabs, Ipswich, MA 01938, USA

[3]glyXera GmbH, 39120 Magdeburg, Germany

[4]Chair of Bioprocess Engineering, Otto-von-Guericke University, 39106 Magdeburg, Germany

*correspondence: taron@neb.com

Supplementary methods

Isolation of human gut microbiome DNA.

Microbial cells were isolated from 100 mg of fecal material with 1 mL Phosphate Buffered Saline (PBS). Sample was centrifuged at 500 x g for 1 min to remove large-sized debris. The supernatant was recovered in a clean tube. To minimize loss of microbial cells, the pellet was washed with 1 mL PBS and centrifuged at 500 x g for 1 min. Pooled supernatants were centrifuged at 3,000 x g for 10 min to pellet microbial cells. Supernatant was discarded. Pelleted cells were washed once with 1 mL PBS followed by centrifugation at 3,000 x g for 10 min. The obtained cell pellet was finally washed one more time with 1 mL of PBS and centrifuged at 500 x g for 1 min to remove big particles. Supernatant containing microbial cells in suspension was saved and centrifuged at 3,000 x g for 10 min to obtain a pellet.

High Performance Anion Exchange Chromatography with Pulsed Amperometric Detection (HPAEC-PAD)

HPAEC-PAD analysis was performed on a Dionex ISC 5000+ with a CarboPac^{TM} PA200 3x50 mm guard column and a CarboPac^{TM} PA200 3x250 mm analytical column (Dionex, Sunnyvale, CA). A 25 µL volume of sample or 25 µM standard monosaccharide dissolved in water was injected using the full loop injection mode. Separation was performed at 30°C using water as eluent A, 1 M sodium acetate in 1 mM NaOH as eluent B and 100 mM NaOH as eluent C and the following gradient: from 0-12min: 90% A - 10% C, from 12-25 min: 70% A - 20% B - 10% C, from 25-28 min: 40% B - 60% C and from 28-39: 40% B - 60% C.

Glycan fractionation

Glycan fractionation was performed on a Dionex Ultimate 3000 HPLC equipped with a Jasco FP-2020+ fluorescence detector using a TSKgel Amide-80 HR column (5 µm particle size, 25 cm x 4.6 mm; length x internal diameter) preceded by a TSKgel Amide-80 Guard (5 µm particle size, 1 cm X 4.6 mm; length x internal diameter). MQ water with 0.2% acetic acid and 0.2% triethylamine was used as solvent A and acetonitrile containing 0.1% acetic acid was used as solvent B. The gradient used for separation

was: 0 min, 80% B; 0-10 min, 70 % B; 10-120 min, 55% B; 120-123 min, 0% B; 130-136 min, 80% B, 136-174, 80% B. Samples were kept at 4°C prior to injection. A volume of 90 µL of sample containing 80% acetonitrile was injected and separation was performed at 30°C. The fluorescence detection wavelengths were: λ_{ex}=448 nm and λ_{em}=510 nm. Fractions were collected from 20 – 70 min by automatic peak detection. Fractions containing *N*-glycans of interest were dried and resuspended with MQ water for use as enzyme substrates.

Primer name	Sequence 5' -> 3'	Use
T7_universal_primer	TAATACGACTCACTATAGGG	Fosmid Sanger sequencing
pCC1_reverse_primer (Lucigen)	CTCGTATGTTGTGTGGAATTGTGAGC	Fosmid Sanger sequencing
PURExpress_F1_ORF13_Fwd	GCGAATTAATACGACTCACTATAGGGCTTAAGTATAAGGAGGAAAAAATATGGTAGGAATTATGTTAATAAAAAAA	*In vitro* expression of ORF17 (sulfatase) from fosmid F1
PURExpress_F1_ORF13_Rev	AAACCCCTCCGTTTAGAGAGGGGTTATGCTAGTTAGTCTTTGTATATATTCCGGATAGG	
PURExpress_F5_ORF2_Fwd	GCG AAT TAA TAC GAC TCA CTA TAG GGC TTA AGT ATA AGG AGG AAA AAA TAT GAG TTA TGT TCC GGG GGT CGC CAA C	*In vitro* expression of ORF2 (sulfatase) from fosmid F5
PURExpress_F5_ORF2_Rev	AAA CCC CTC CGT TTA GAG AGG GGT TAT GCT AGT TAC CGG CGA ACA GCG AAT CGA AGA AA	
PURExpress_F6_ORF7_Fwd	GCG AAT TAA TAC GAC TCA CTA TAG GGC TTA AGT ATA AGG AGG AAA AAA TAT GGA ACA TCA AAA CAA ATT GAT TTA T	*In vitro* expression of ORF7 (sulfatase) from fosmid F6
PURExpress_F6_ORF7_Rev	AAA CCC CTC CGT TTA GAG AGG GGT TAT GCT AGT TAT TTT ACA AAG TCT GTA TCG TTA TA	
PURExpress_F6_ORF9_Fwd	GCG AAT TAA TAC GAC TCA CTA TAG GGC TTA AGT ATA AGG AGG AAA AAA TAT GGC CGG TAG CCT TGG TTT GTC CGC T	*In vitro* expression of ORF9 (sulfatase) from fosmid F6
PURExpress_F6_ORF9_Rev	AAA CCC CTC CGT TTA GAG AGG GGT TAT GCT AGT TAT TTT TTA TTT GTT GGA TAA TTC GG	
PURExpress_F8_ORF12 _Fwd	GCG AAT TAA TAC GAC TCA CTA TAG GGC TTA AGT ATA AGG AGG AAA AAA TAT GAA ACA AAC AGT TAT AGC TTT AGG A	*In vitro* expression of ORF12 (sulfatase) from fosmid F8
PURExpress_F8_ORF12_Rev	AAA CCC CTC CGT TTA GAG AGG GGT TAT GCT AGT TAT AAG GTT TCC ATG TTA GAA AGT AA	
PURExpress_F8_ORF16_Fwd	GCG AAT TAA TAC GAC TCA CTA TAG GGC TTA AGT ATA AGG AGG AAA AAA TAT GAA AAA CTT ACA ATC AGG ATT ACT C	*In vitro* expression of ORF16 (sulfatase) from fosmid F8
PURExpress_F8_ORF16_Rev	AAA CCC CTC CGT TTA GAG AGG GGT TAT GCT AGT TAT TCT TTA TCT CTC TCT GGG GAA AA	
PURExpress_F10_ORF22_Fwd	GCG AAT TAA TAC GAC TCA CTA TAG GGC TTA AGT ATA AGG AGG AAA AAA TAT GAA TTA CAA ATC TAT ATC ATT AAT A	*In vitro* expression of ORF22 (sulfatase) from fosmid F10
PURExpress_F10_ORF22_Rev	AAA CCC CTC CGT TTA GAG AGG GGT TAT GCT AGT TAC TGC TTA GGC AGT GTC ACA GAA AA	
PURExpress_F10_ORF23_Fwd	GCG AAT TAA TAC GAC TCA CTA TAG GGC TTA AGT ATA AGG AGG AAA AAA TAT GAA ACA ACC TTT GCT TTT TAC CCT T	*In vitro* expression of ORF23 (sulfatase) from fosmid F10
PURExpress_F10_ORF23_Rev	AAA CCC CTC CGT TTA GAG AGG GGT TAT GCT AGT TAT TTG CCG ATA AGG TCT TTT CCT TC	
PURExpress_F3_ORF26_Fwd	GCG AAT TAA TAC GAC TCA CTA TAG GGC TTA AGT ATA AGG AGG AAA AAA TAT GAA AAA ACA ACT GAT GCA ATG GGC A	*In vitro* expression of ORF26 (hexosaminidase) from fosmid F3
PURExpress_F3_ORF26_Rev	AAA CCC CTC CGT TTA GAG AGG GGT TAT GCT AGT TAC TCC ACT CCT ATT TCG TCA ACA AA	
PURExpress_ F10_ORF19_Fwd	GCG AAT TAA TAC GAC TCA CTA TAG GGC TTA AGT ATA AGG AGG AAA AAA TAT GAA AAA CAA GTA TCT TTT ATC	*In vitro* expression of ORF19 (hexosaminidase) from fosmid F10
PURExpress_F10_ORF19_Rev	AAA CCC CTC CGT TTA GAG AGG GGT TAT GCT AGT TAC TTC ATT ATA TGT TTG CCG TAA TT	
ORF19_hex_pETover_Fwd	CTTTAAGAAGGAGATATACCATGCAAGAAATAGCTATCATTCCGC	Cloning ORF19 (hexosaminidase) from fosmid F10 into pET28c(+)_B1006 without its native signal sequence and with a 6 x His C-term tag.
ORF19_hex_pETover_Rev	CAGTGGTGGTGGTGGTGGTGCTTCATTATATGTTTGCCGTAATTCC	
pET28c(+)_ORF19overl_Fwd	ACGGCAAACATATAATGAAGCACCACCACCACCACCACTGAGATC	Linearization of pET28c(+)_B1006 vector for HiFi cloning of ORF19 hexosmainidase.
pET28c(+)_ORF19overl_Rev	ATGATAGCTATTTCTTGCATGGTATATCTCCTTCTTAAAGTTAAACAAAATTATTTCTAGAGGGGAATTGTTATC	
pET21a(+)_ORF17_6His_Fwd	AACCTGAACTATTTATATAAAGAAATAATTTTGTTTAACTTTA	Linearization of pET21a(+) vector for HiFi cloning of ORF2
pET21a(+)_ORF17_6His_Rev	TGCCCATTGTATATCTCCTTAGAGGGGAATTGTTATCC	
RBS_F1_ORF2_Fwd	aaggagatatacaATGGGCAAAAGAATAGAAAT	Cloning ORF2 from fosmid F1 with an upstream RBS, for insertion into pET21a(+)_ORF17
F1_ORF2_Rev	TTATATAAATAGTTCAGGTTCACGGC	

Table S1. Primer sequences used in this study

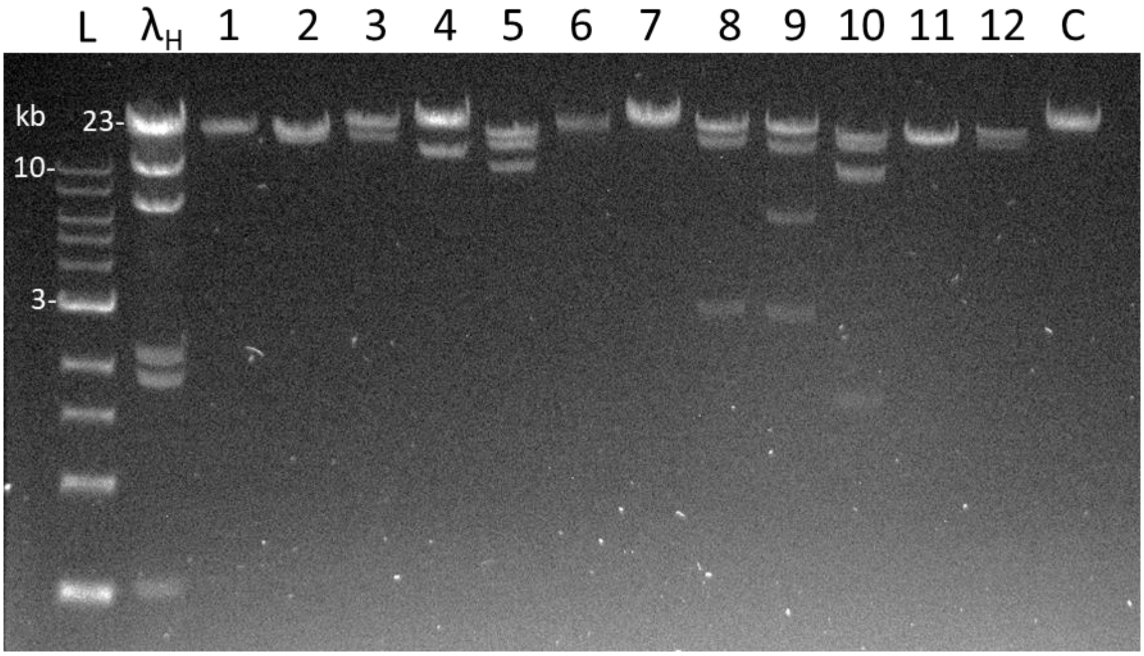

Figure S1. Restriction fragment analysis of 12 clones from the human gut metagenomics library.

The 12 fosmids were digested with the enzyme Fse I (1-12). Two ladders were used: 1kb Plus DNA Ladder (L) and lambda DNA digested with Hind III (λ_H). A 40 kb fosmid control was also run to serve as a size marker (C).

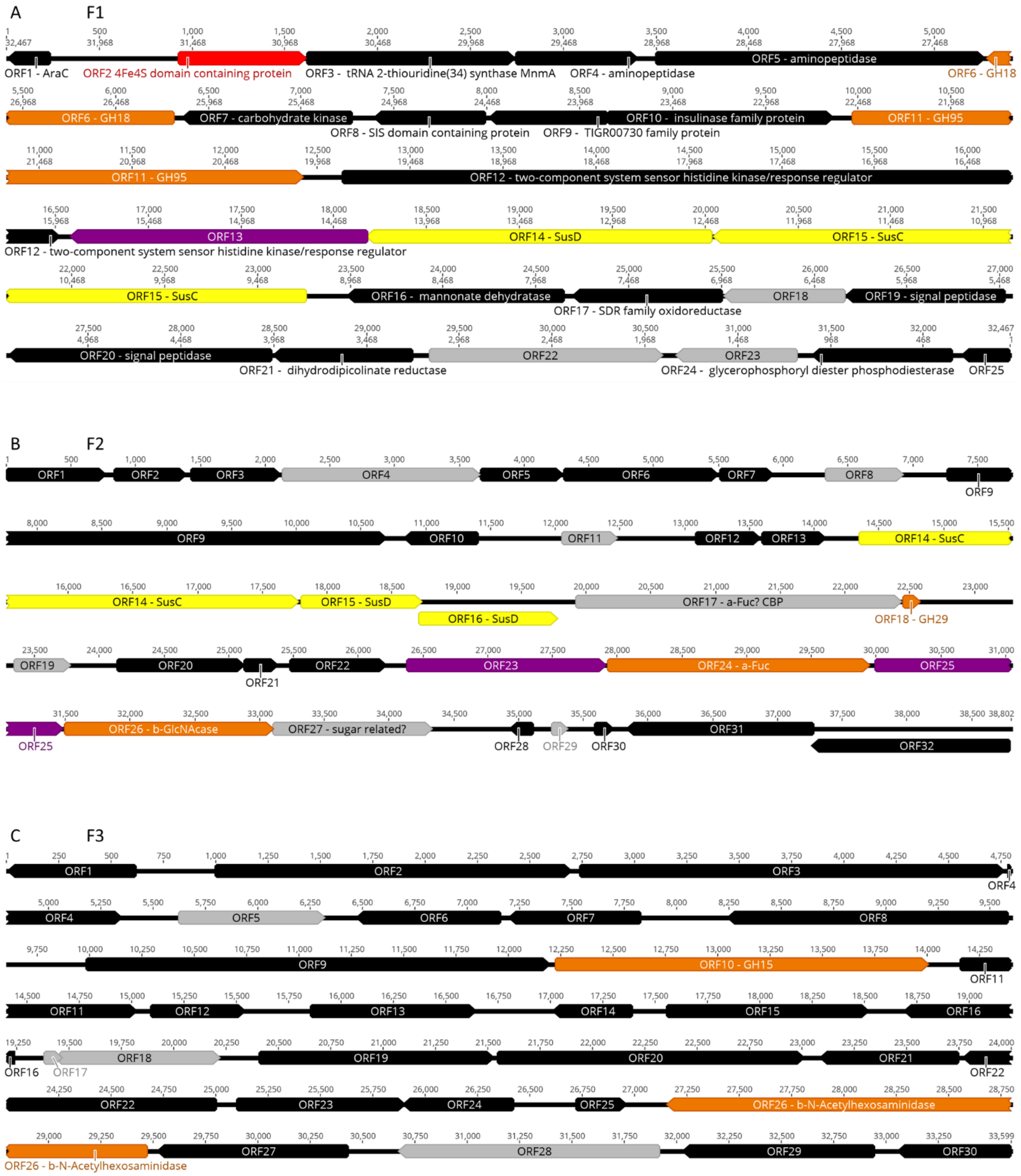
A
F1
ORF1 - AraC
ORF2 4Fe4S domain containing protein
ORF3 - tRNA 2-thiouridine(34) synthase MnmA
ORF4 - aminopeptidase
ORF5 - aminopeptidase
ORF6 - GH18
ORF7 - carbohydrate kinase
ORF8 - SIS domain containing protein
ORF9 - TIGR00730 family protein
ORF10 - insulinase family protein
ORF11 - GH95
ORF12 - two-component system sensor histidine kinase/response regulator
ORF13
ORF14 - SusD
ORF15 - SusC
ORF16 - mannonate dehydratase
ORF17 - SDR family oxidoreductase
ORF18
ORF19 - signal peptidase
ORF20 - signal peptidase
ORF21 - dihydrodipicolinate reductase
ORF22
ORF23
ORF24 - glycerophosphoryl diester phosphodiesterase
ORF25
B
F2
ORF1
ORF2
ORF3
ORF4
ORF5
ORF6
ORF7
ORF8
ORF9
ORF10
ORF11
ORF12
ORF13
ORF14 - SusC
ORF15 - SusD
ORF16 - SusD
ORF17 - a-Fuc? CBP
ORF18 - GH29
ORF19
ORF20
ORF21
ORF22
ORF23
ORF24 - a-Fuc
ORF25
ORF26 - b-GlcNAcase
ORF27 - sugar related?
ORF28
ORF29
ORF30
ORF31
ORF32
C
F3
ORF1
ORF2
ORF3
ORF4
ORF5
ORF6
ORF7
ORF8
ORF9
ORF10 - GH15
ORF11
ORF12
ORF13
ORF14
ORF15
ORF16
ORF17
ORF18
ORF19
ORF20
ORF21
ORF22
ORF23
ORF24
ORF25
ORF26 - b-N-Acetylhexosaminidase
ORF27
ORF28
ORF29
ORF30

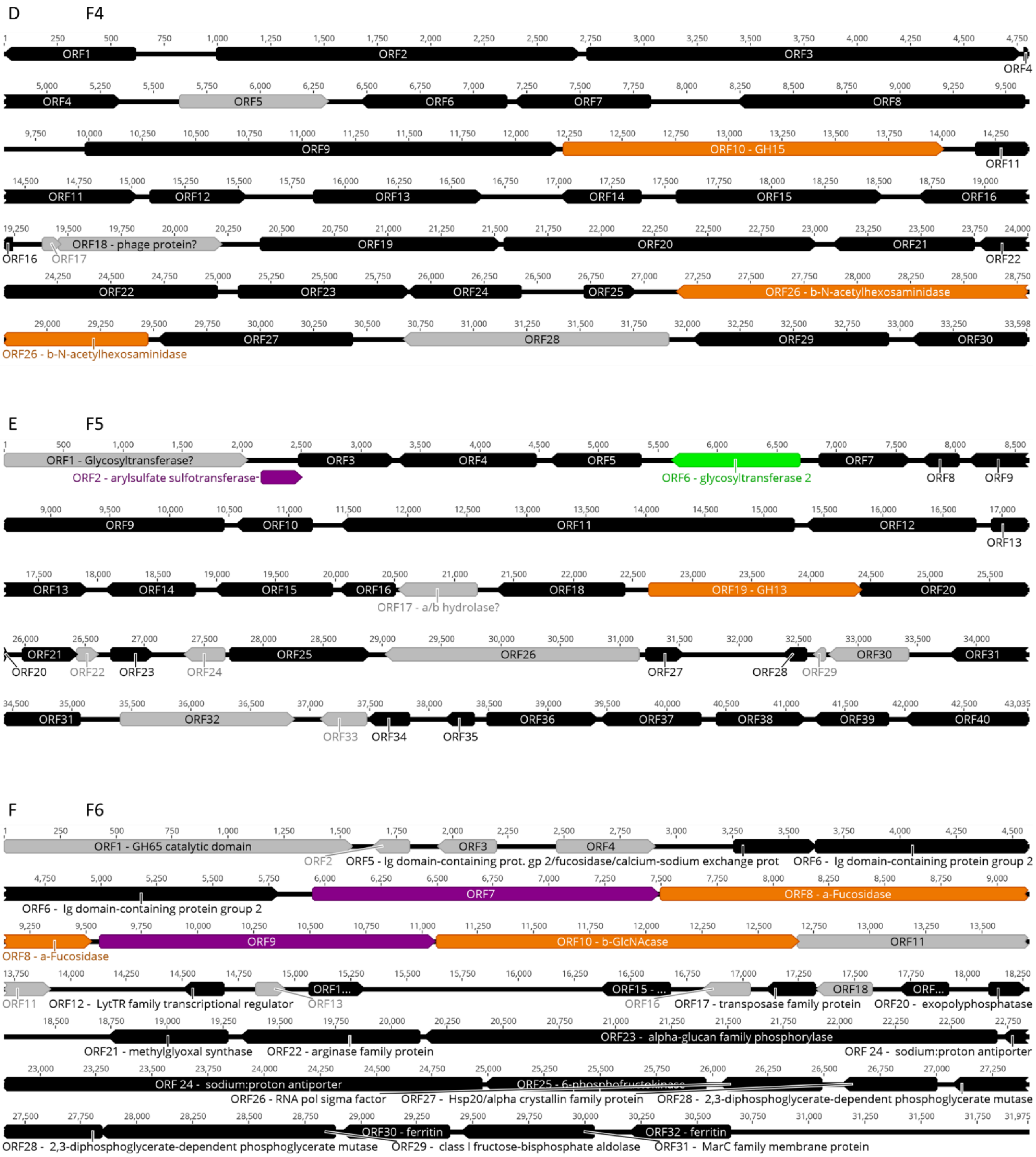

D
F4
ORF1
ORF2
ORF3
ORF4
ORF5
ORF6
ORF7
ORF8
ORF9
ORF10 - GH15
ORF11
ORF12
ORF13
ORF14
ORF15
ORF16
ORF17
ORF18 - phage protein?
ORF19
ORF20
ORF21
ORF22
ORF23
ORF24
ORF25
ORF26 - b-N-acetylhexosaminidase
ORF27
ORF28
ORF29
ORF30
E
F5
ORF1 - Glycosyltransferase?
ORF2 - arylsulfate sulfotransferase
ORF3
ORF4
ORF5
ORF6 - glycosyltransferase 2
ORF7
ORF8
ORF9
ORF10
ORF11
ORF12
ORF13
ORF14
ORF15
ORF16
ORF17 - a/b hydrolase?
ORF18
ORF19 - GH13
ORF20
ORF21
ORF22
ORF23
ORF24
ORF25
ORF26
ORF27
ORF28
ORF29
ORF30
ORF31
ORF32
ORF33
ORF34
ORF35
ORF36
ORF37
ORF38
ORF39
ORF40
F
F6
ORF1 - GH65 catalytic domain
ORF2
ORF3
ORF4
ORF5 - Ig domain-containing prot. gp 2/fucosidase/calcium-sodium exchange prot
ORF6 - Ig domain-containing protein group 2
ORF7
ORF8 - a-Fucosidase
ORF9
ORF10 - b-GlcNAcase
ORF11
ORF12 - LytTR family transcriptional regulator
ORF13
ORF16
ORF17 - transposase family protein
ORF18
ORF20 - exopolyphosphatase
ORF21 - methylglyoxal synthase
ORF22 - arginase family protein
ORF23 - alpha-glucan family phosphorylase
ORF 24 - sodium:proton antiporter
ORF25 - 6-phosphofructokinase
ORF26 - RNA pol sigma factor
ORF27 - Hsp20/alpha crystallin family protein
ORF28 - 2,3-diphosphoglycerate-dependent phosphoglycerate mutase
ORF29 - class I fructose-bisphosphate aldolase
ORF30 - ferritin
ORF31 - MarC family membrane protein
ORF32 - ferritin

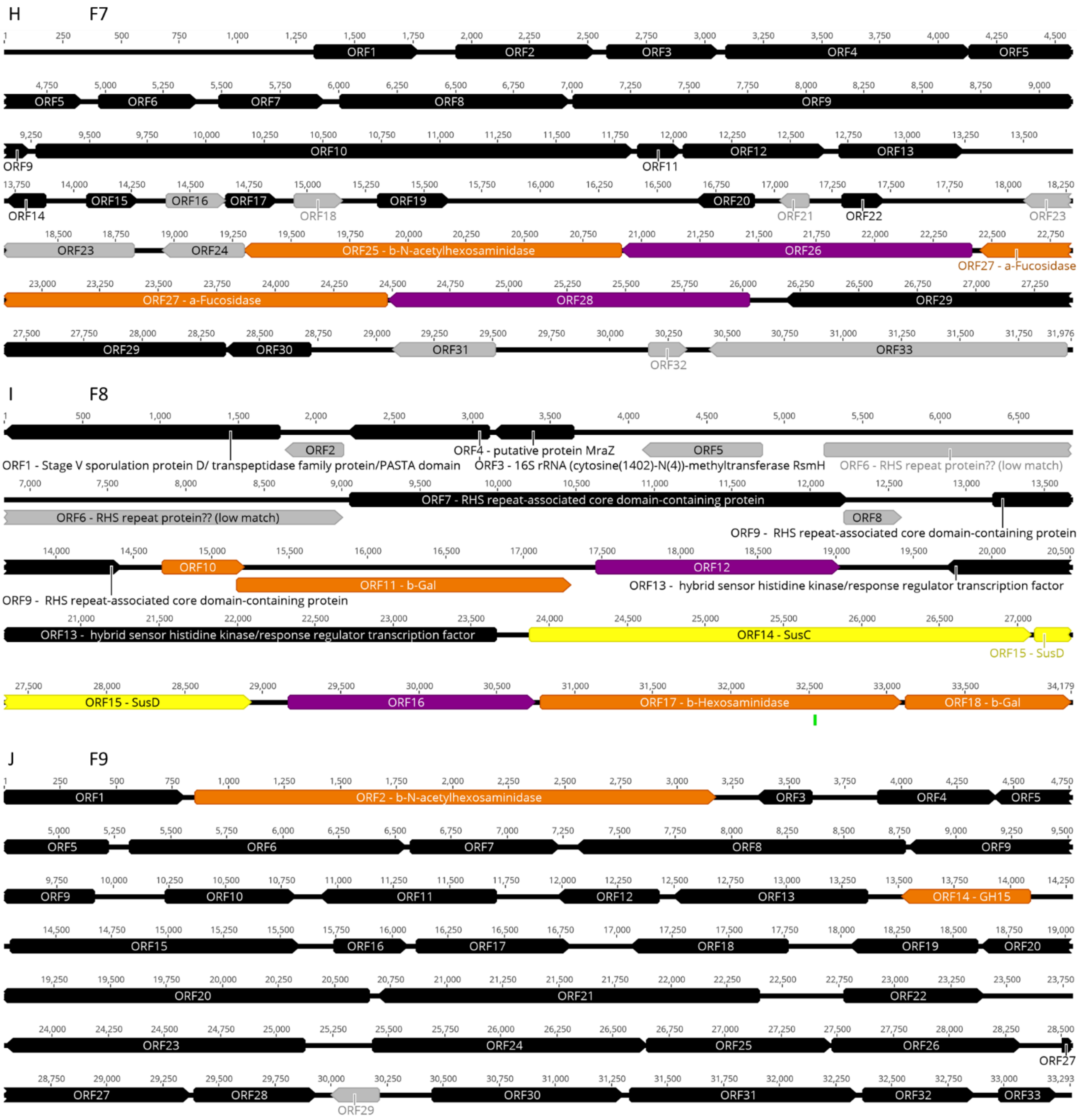
H
F7
ORF1
ORF2
ORF3
ORF4
ORF5
ORF6
ORF7
ORF8
ORF9
ORF10
ORF11
ORF12
ORF13
ORF14
ORF15
ORF16
ORF17
ORF18
ORF19
ORF20
ORF21
ORF22
ORF23
ORF24
ORF25 - b-N-acetylhexosaminidase
ORF26
ORF27 - a-Fucosidase
ORF28
ORF29
ORF30
ORF31
ORF32
ORF33
I
F8
ORF1 - Stage V sporulation protein D/ transpeptidase family protein/PASTA domain
ORF2
ORF3 - 16S rRNA (cytosine(1402)-N(4))-methyltransferase RsmH
ORF4 - putative protein MraZ
ORF5
ORF6 - RHS repeat protein?? (low match)
ORF7 - RHS repeat-associated core domain-containing protein
ORF8
ORF9 - RHS repeat-associated core domain-containing protein
ORF10
ORF11 - b-Gal
ORF12
ORF13 - hybrid sensor histidine kinase/response regulator transcription factor
ORF14 - SusC
ORF15 - SusD
ORF16
ORF17 - b-Hexosaminidase
ORF18 - b-Gal
J
F9
ORF1
ORF2 - b-N-acetylhexosaminidase
ORF3
ORF4
ORF5
ORF6
ORF7
ORF8
ORF9
ORF10
ORF11
ORF12
ORF13
ORF14 - GH15
ORF15
ORF16
ORF17
ORF18
ORF19
ORF20
ORF21
ORF22
ORF23
ORF24
ORF25
ORF26
ORF27
ORF28
ORF29
ORF30
ORF31
ORF32
ORF33

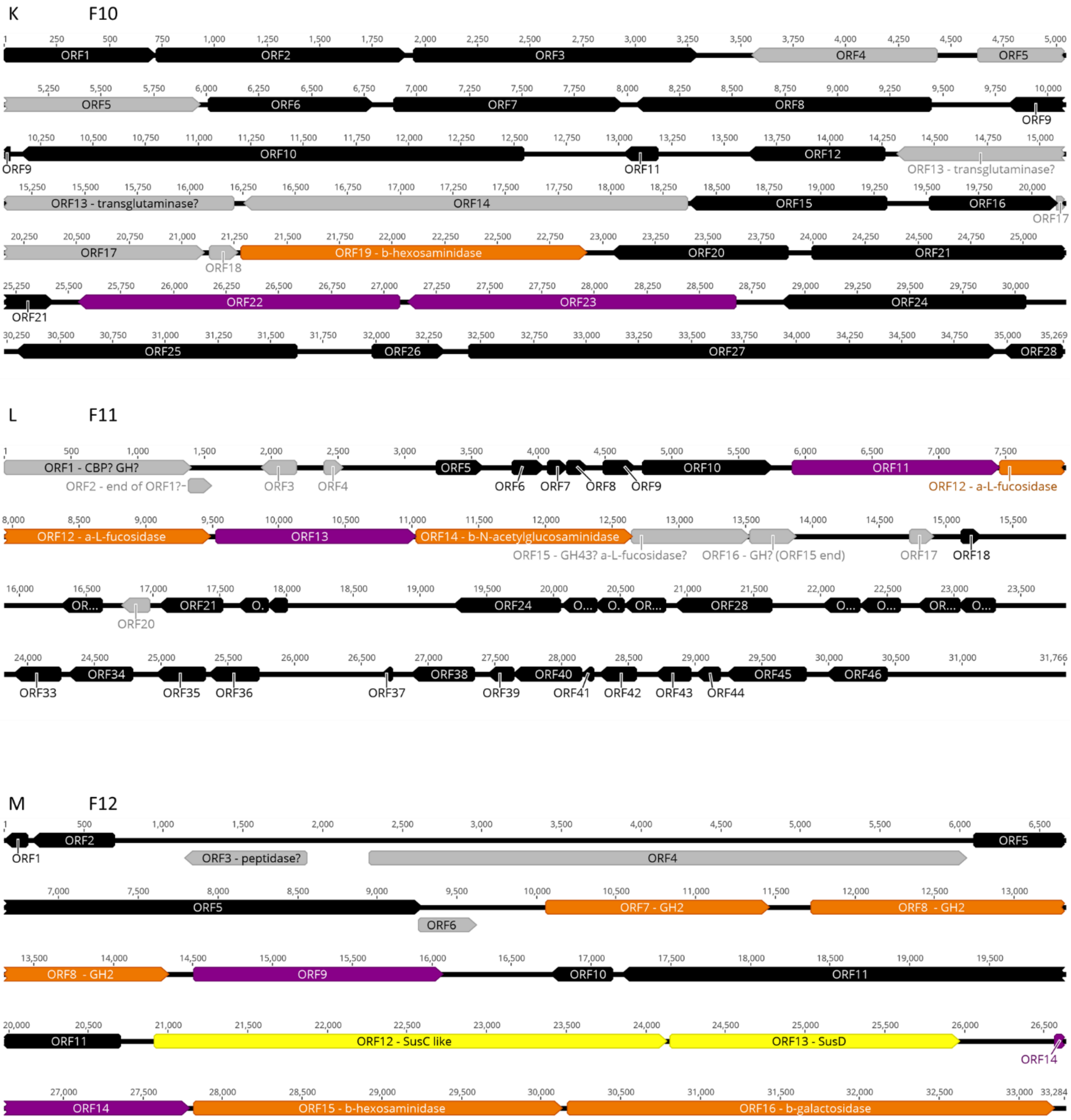

K
F10
ORF1
ORF2
ORF3
ORF4
ORF5
ORF6
ORF7
ORF8
ORF9
ORF10
ORF11
ORF12
ORF13 - transglutaminase?
ORF14
ORF15
ORF16
ORF17
ORF18
ORF19 - b-hexosaminidase
ORF20
ORF21
ORF22
ORF23
ORF24
ORF25
ORF26
ORF27
ORF28
L
F11
ORF1 - CBP? GH?
ORF2 - end of ORF1?
ORF12 - a-L-fucosidase
ORF13
ORF14 - b-N-acetylglucosaminidase
ORF15 - GH43? a-L-fucosidase?
ORF16 - GH? (ORF15 end)
M
F12
ORF3 - peptidase?
ORF7 - GH2
ORF8 - GH2
ORF12 - SusC like
ORF13 - SusD
ORF15 - b-hexosaminidase
ORF16 - b-galactosidase

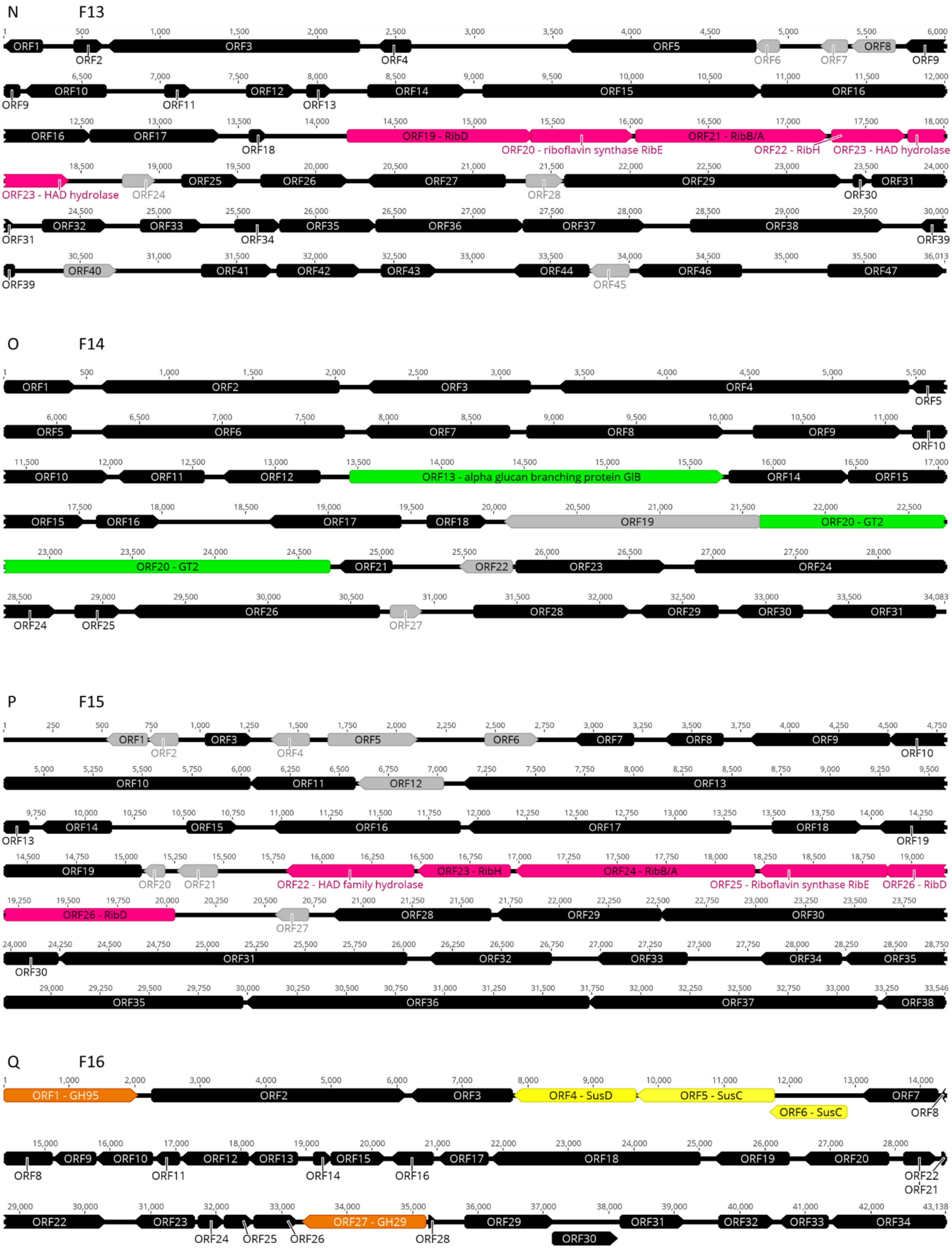
N
F13
ORF19 - RibD
ORF20 - riboflavin synthase RibE
ORF21 - RibB/A
ORF22 - RibH
ORF23 - HAD hydrolase
O
F14
ORF13 - alpha glucan branching protein GIB
ORF20 - GT2
P
F15
ORF22 - HAD family hydrolase
ORF23 - RibH
ORF24 - RibB/A
ORF25 - Riboflavin synthase RibE
ORF26 - RibD
Q
F16
ORF1 - GH95
ORF4 - SusD
ORF5 - SusC
ORF6 - SusC
ORF27 - GH29

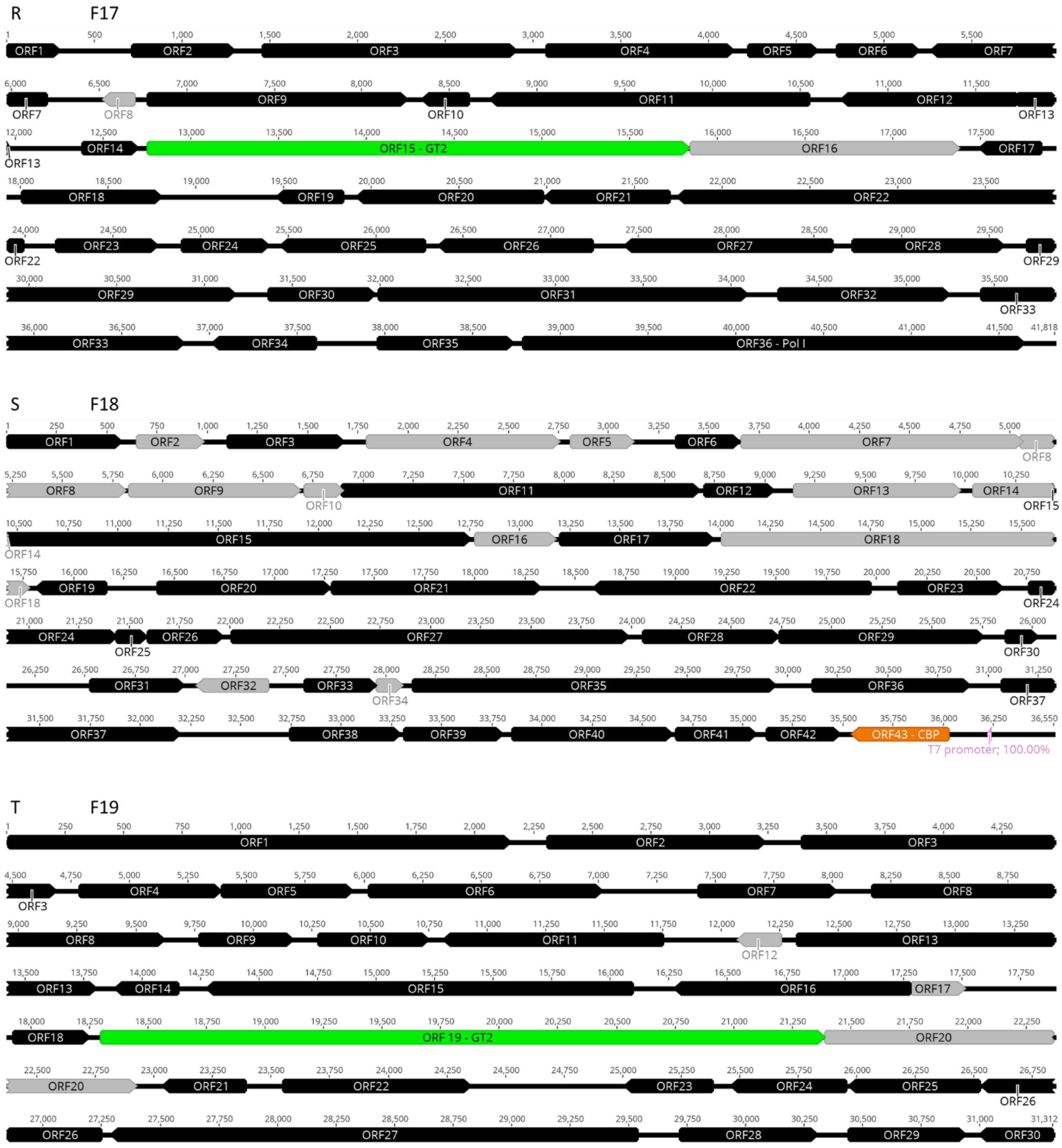

Figure S2. Open reading frame (ORF) maps.

An ORF map for 19 hits was produced using Geneious (https://www.geneious.com). ORFs that encode for sulfatases are colored in **purple**, ORFs that encode for glycoside hydrolase including hexosaminidases are colored in **orange**, ORFs that encode for the sugar transporter SusC/D pair are colored in **yellow**, ORFs that encode for glycosyl transferase are colored in **green**, ORFs belonging to the riboflavin biosynthesis pathway are cloroed in **pink**, ORFs in **black** are ORFs with significant match to protein of known function while ORFs with very little to no homology to known protein are represented in **grey**. ORF2 from F1 (A) is colored in **red** as assignment of its function was attempted in this work.

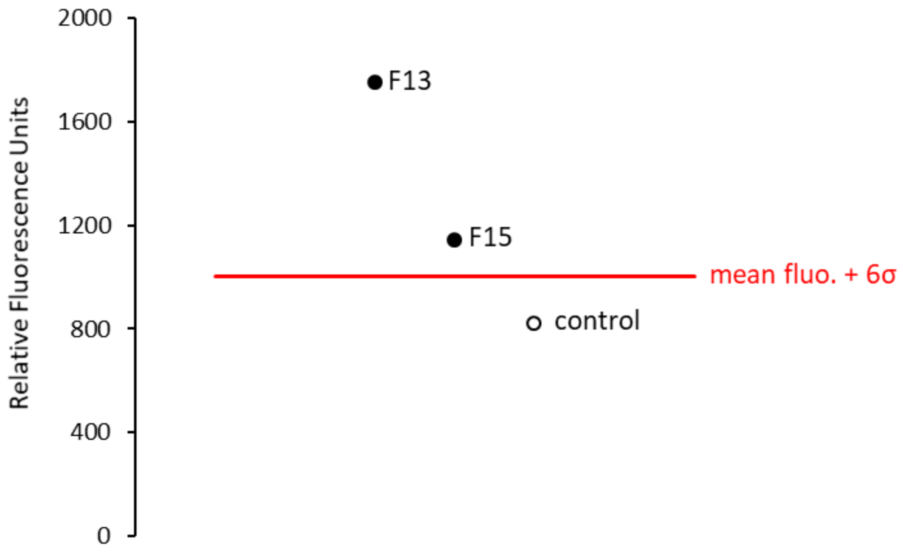

Figure S3. Background fluorescence of F13 and F15.

Fluorescence at 365/445 nm was measured for F13 and F15 in absence of 4MU-substrate. Fluorescence generated by both clones is higher than the control and higher than 6 standard deviation above the mean (red line).

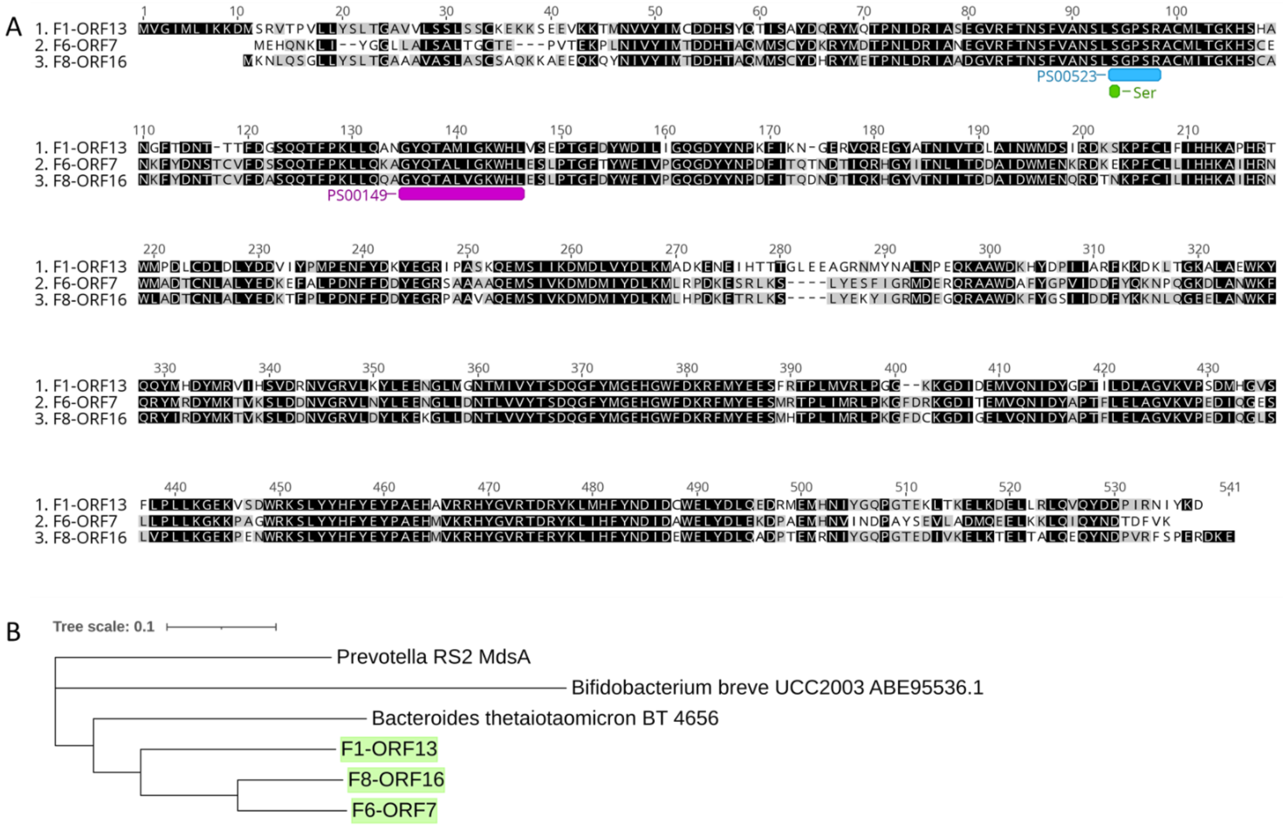

Figure S4. ORF7, 13 and 16 relationships to other GlcNAc-6-sulfatases

A. F6-ORF7, F1-ORF13 and F8-ORF16 protein sequence alignment using the BLOSSUM62 score matrix. All three protein have a high sequence similarity. They belong to the family S1 formylglycine-dependent sulfatases. The consensus motifs from this family PS00149 and PS00523 are annotated in purple and blue respectively. The critical catalytic residue Ser is highlighted in green. **B. Phylogenetic tree of members of the family S1 sulfatase. Protein** highlighted in green were identified in this work.

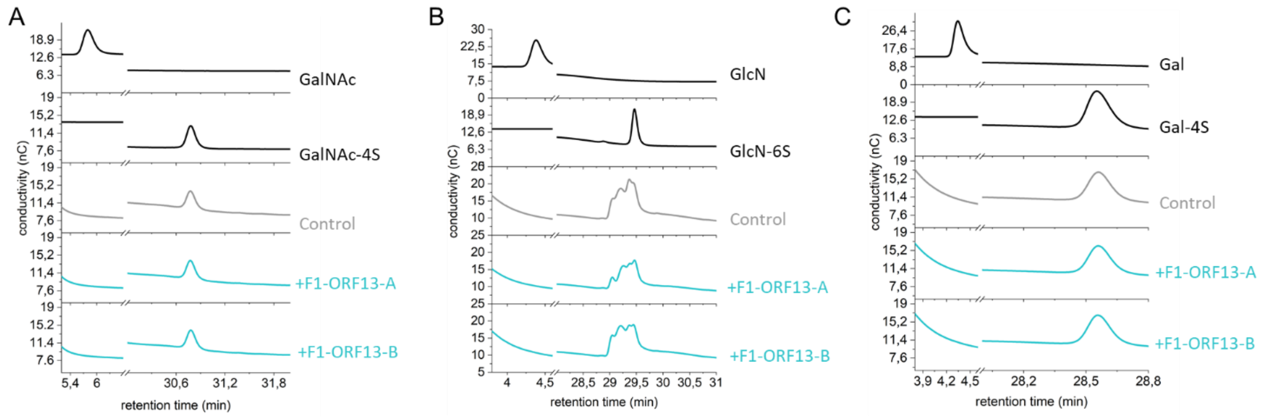

Figure S5. F1-ORF13 substrate specificity

A, B and C. Activity of *in vitro* expressed F1-ORF13 sulfatase on GalNAc-4-SO_4 (A), GlcN-6-SO_4 (B) and Gal-4-SO_4 (C). Reactions were performed in duplicates (F1-ORF13-A and -B – blue chromatograms). Control consists of *in vitro* expression mixture devoid of F1-ORF13 (grey chromatogram). Sulfated and non-sulfated monosaccharides dissolved in water were run as standards for retention time comparison with reaction samples (black chromatograms). Analysis was performed by high-performance anion-exchange chromatography with pulse amperometric detection (HPAEC-PAD) (see material and methods for details).

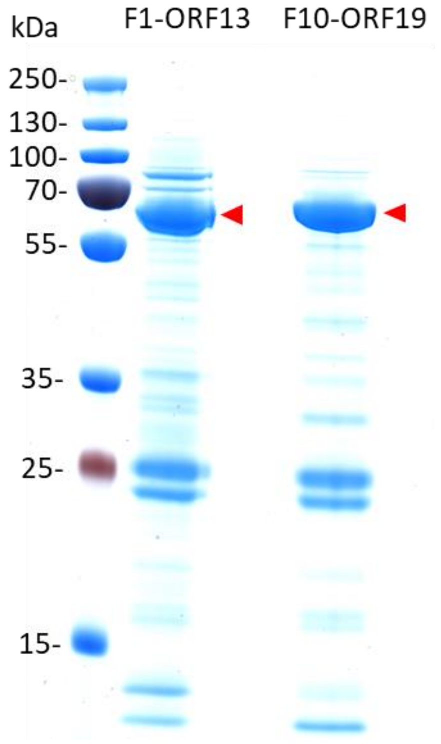

Figure S6. Partially purified F1-ORF13 and F10-ORF19

F1-ORF13 sulfatase and F10-ORF19 hexosaminidase were over-expressed in *E. coli* and partially purified on a His-Trap column.

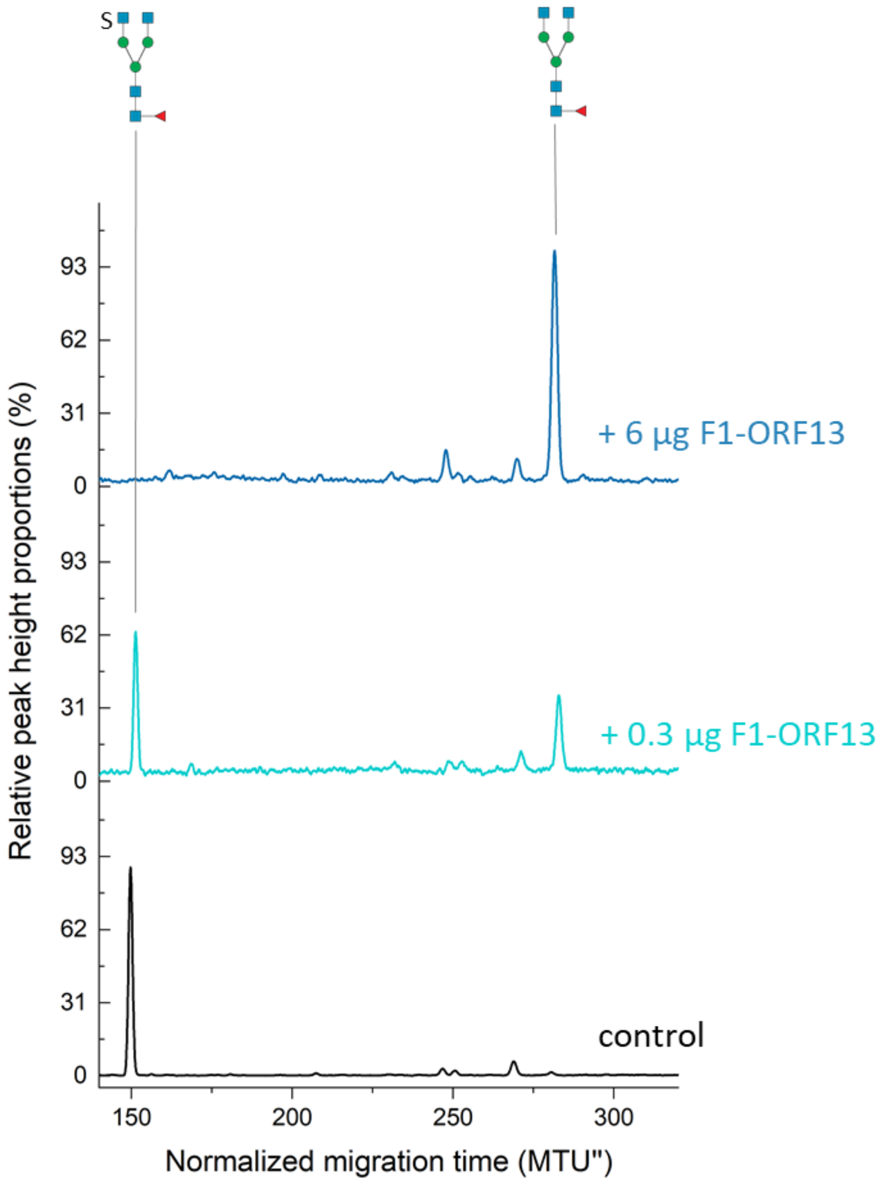

Figure S7. F1-ORF13 activity on FA2G0-SO_4 is concentration dependent.

F1-ORF13 was assayed on an APTS-labelled *N*-glycan substrate (termed FA2G0-SO_4) generated from human immunoglobulin A. Substrate and product were monitored by xCGE-LIF. The x-axis (time) of the electropherograms was normalized to two internal standards by glyXtool^{CE}, resulting in double normalized migration time units (MTU’’). Signal Intensities were normalized to the total peak height, resulting in relative peak height proportions (%).. Glycans are represented using the SNFG nomenclature [3].

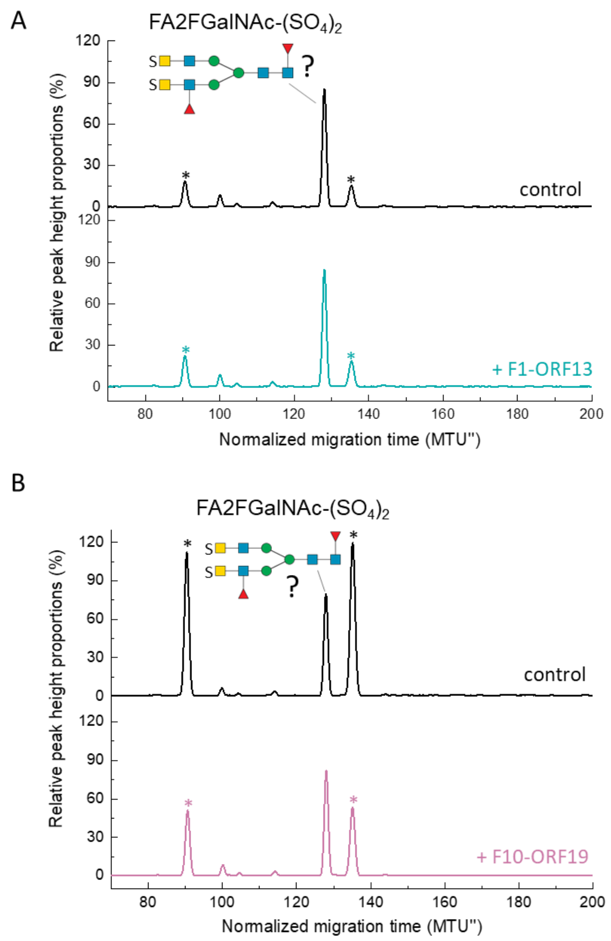

Figure S8. F1-ORF13 and F10-ORF19 activities on sulfated GalNAc-containing *N*-glycan.

A. F1-ORF13 sulfatase activity on an APTS-labelled *N*-glycan isolated from human urokinase. A urokinase APTS-labelled *N*-glycan containing two terminal sulfated GalNAc residues (FA2FGalNAc-$(SO_4)_2$) was used as a substrate to test F1-ORF13 sulfatase (black electropherogram). The exact structure of the substrate is marked with (?) as assigned based on the literature [1, 2] and not fully experimentally confirmed. No activity could be detected as shown with the absence of migration of the peak correspond to the substrate (blue electropherogram). **B. F10-ORF19 hexosaminidase activity on a urokinase isolated *N*-glycan.** The same substrate was used to test F10-ORF19 hexosaminidase (black electropherogram). No activity could be detected as shown with the absence of migration of the peak correspond to the substrate (pink electropherogram). The x-axis (time) of the electropherograms was normalized to two internal standards by glyXtool^{CE}, resulting in double normalized migration time units (MTU''). Signal Intensities were normalized to the total peak height, resulting in relative peak height proportions (%). Peaks marked with (*) correspond to the internal standard used for migration time normalization in xCGE-LIF. Glycans are represented using the SNFG nomenclature [3].

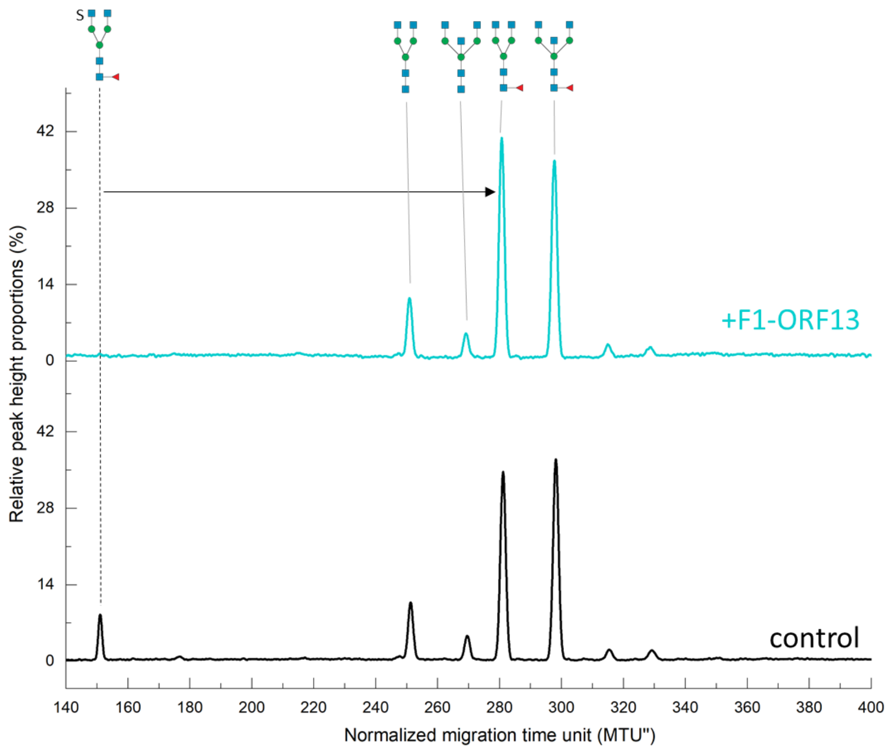

Figure S9. F1-ORF13 activity on a pool of *N*-glycans.

F1-ORF13 was assayed on a pool of human APTS-labelled immunoglobulin A (hIgA) *N*-glycans pretreated with sialidase and β-galactosidase (black electropherogram). Substrate and product were analyzed by xCGE-LIF. The activity of F1-ORF13 (blue electropherogram) is indicated with a black arrow that shows the migration time shift of the FA2G0-SO_4 peak from ~150 MTU'' to the FA2G0 peak at ~280 MTU'' due to the loss of the sulfate group. The x-axis (time) of the electropherograms was normalized to two internal standards by glyXtool^{CE}, resulting in double normalized migration time units (MTU''). Signal Intensities were normalized to the total peak height, resulting in relative peak height proportions (%).Glycans are represented using the SNFG nomenclature [3].

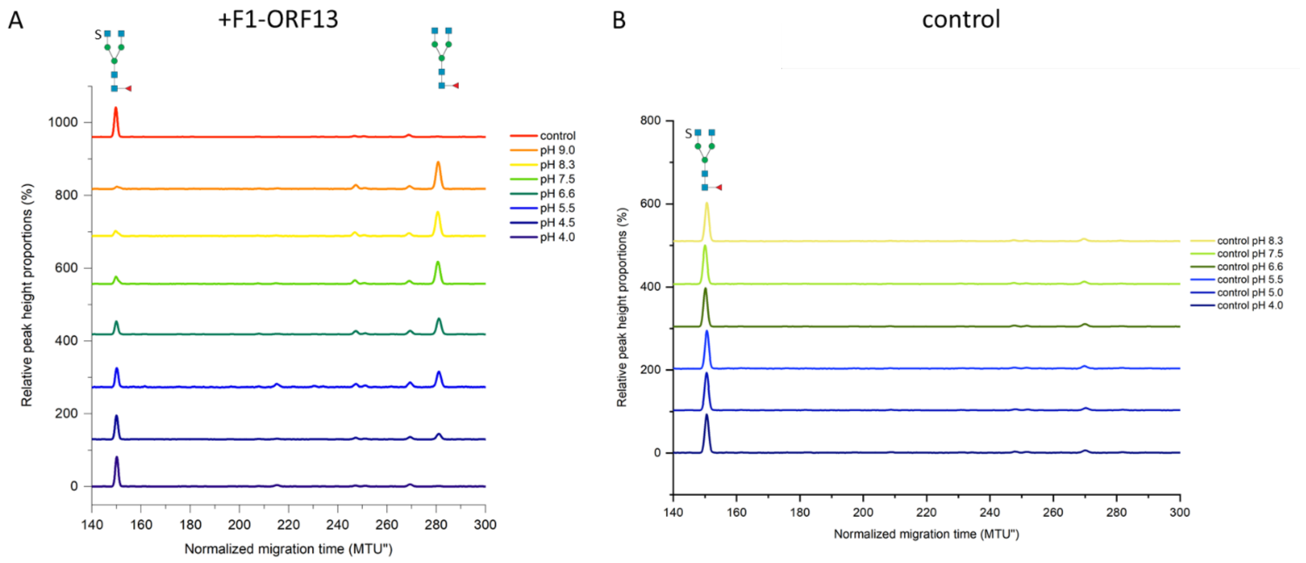

Figure S10. F1-ORF13 activity on FA2G0-SO_4 at a range of pH values.

A. F1-ORF13 sulfatase activity on an APTS-labelled *N*-glycan isolated from human immunoglobulin A. Activity of F1-ORF13 at a pH range of 4-9 was determined using APTS-labelled FA2G0-SO_4 and following product formation by xCGE-LIF. Reactions were performed in triplicates, only one set of experiment is shown. **B. FA2G0-SO_4 stability at a range of pH values.** Integrity of the FA2G0-SO_4 substrate in absence of enzyme at a range of pH values was monitored. The x-axis (time) of the electropherograms was normalized to two internal standards by glyXtool^{CE}, resulting in double normalized migration time units (MTU''). Signal Intensities were normalized to the total peak height, resulting in relative peak height proportions (%). Glycans are represented using the SNFG nomenclature [3].

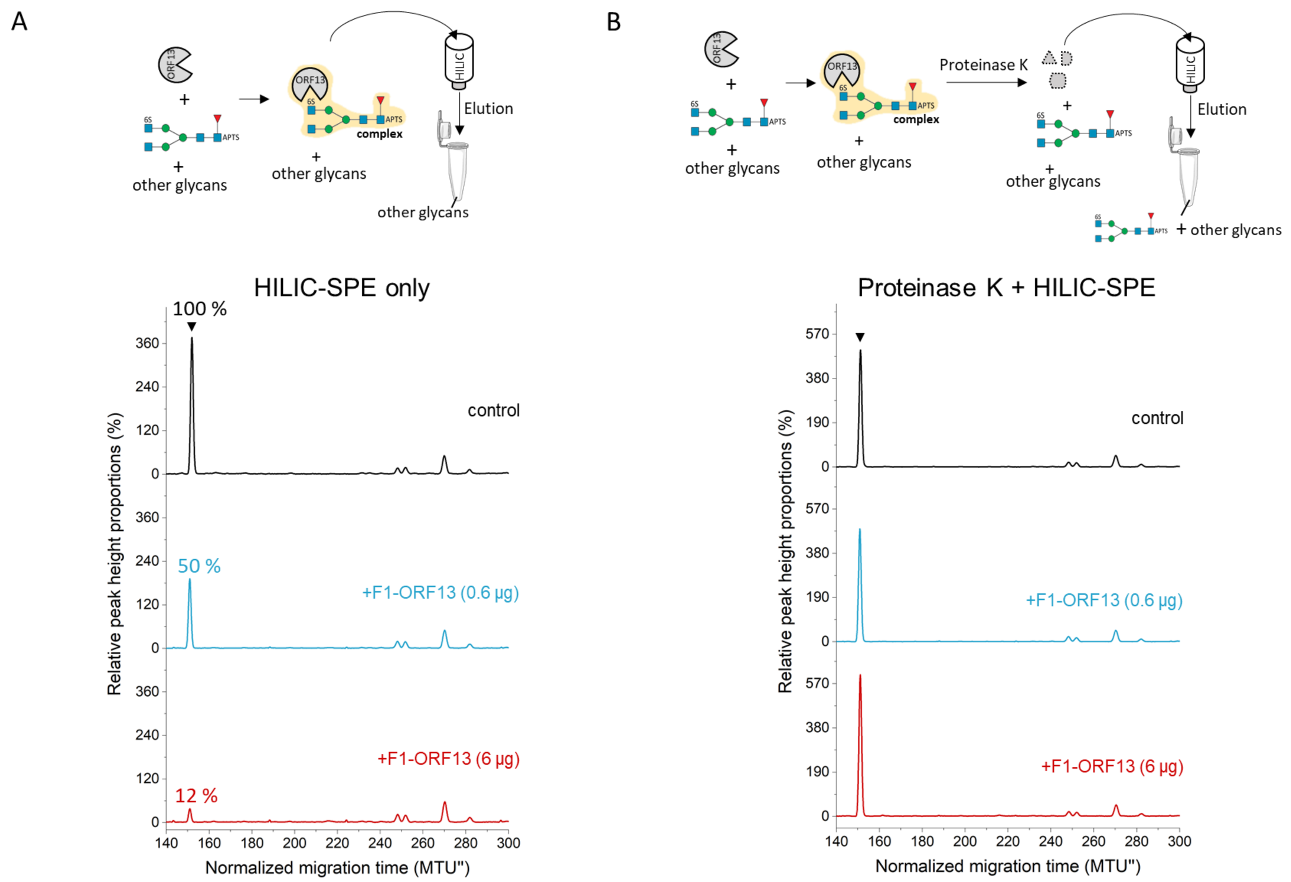

Figure S11. F1-ORF13 activity in absence of calcium

F1-ORF13 activity on an APTS-labelled *N*-glycan FA2G0-SO_4 (black triangle) was evaluated in absence of its cofactor. **A. HILIC-SPE cleaned reactions analyzed by xCGE-LIF.** Following reaction, samples were directly cleaned-up by HILIC-SPE for subsequent analysis by xCGE-LIF. **B. Proteolyzed and HILIC-SPE cleaned reactions analyzed by xCGE-LIF.** Following reaction, samples were treated with proteinase K to destroy F1-ORF13 before clean-up by HILIC-SPE and analysis by xCGE-LIF. The x-axis (time) of the electropherograms was normalized to two internal standards by glyXtool^{CE}, resulting in double normalized migration time units (MTU''). Signal Intensities were normalized to the height of the peaks from 240-285 MTU'', resulting in relative peak height proportions (%). Glycans are represented using the SNFG nomenclature [3].

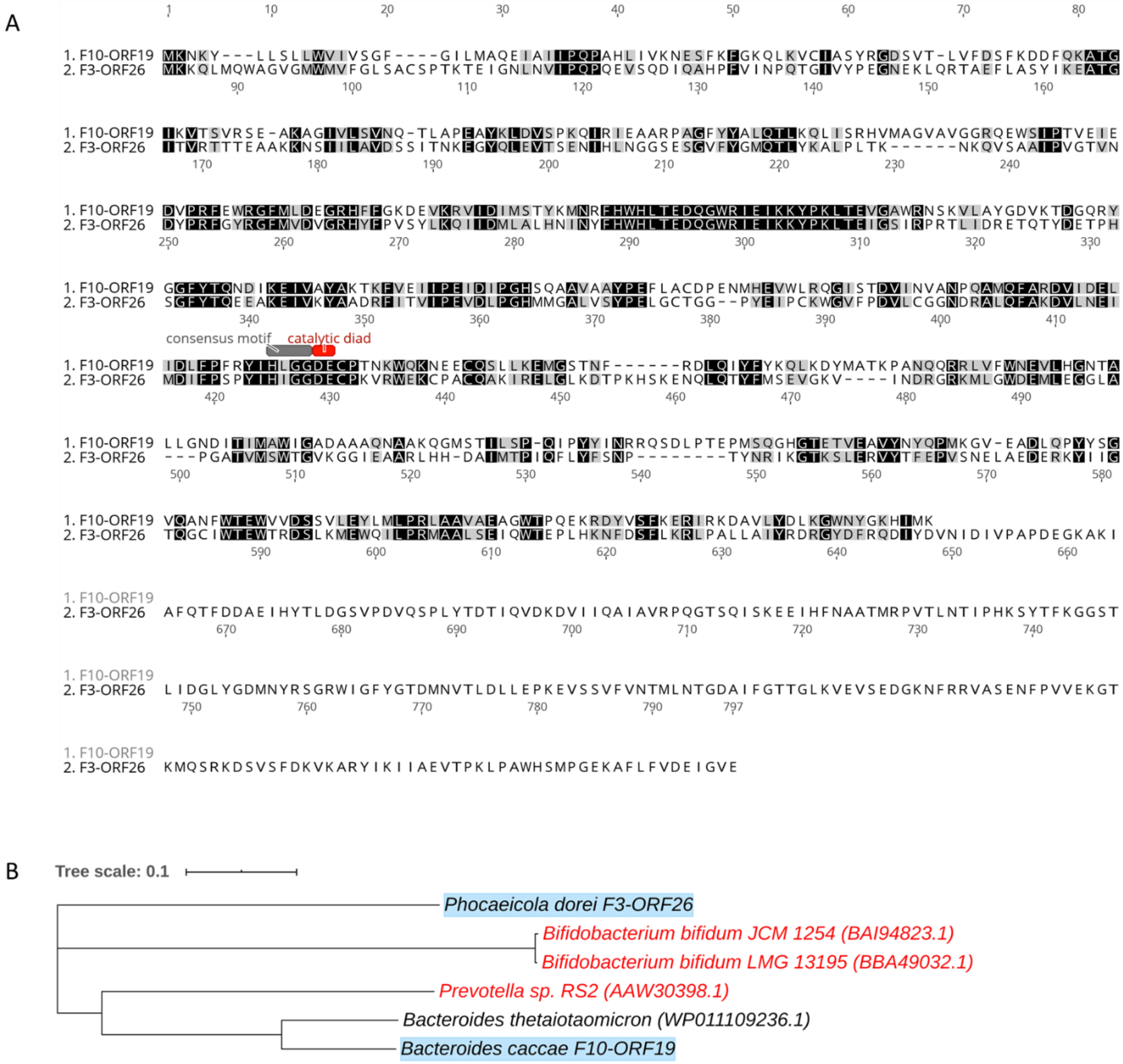

Figure S12. F10-ORF19 and F3-ORF26 are members of glycoside hydrolase family 20 (GH20)

A. F10-ORF19 and F3-ORF26 protein sequence alignment using the BLOSSUM62 score matrix. Both proteins are members of the GH20 family and contain the H-X-G-G consensus motif of this family (grey box). Catalytic diad formed with amino acid D-E is annotated in red. **B. Phylogenetic tree of GH20 members.** Proteins in red were characterized in other studies and their ability to hydrolyze sulfated GlcNAc formerly demonstrated [4, 5]. Protein highlighted in blue were identified in this work.

1. Bergweff AA, Thomas-Oates JE, van Oostrum J, Kamerling JP, Vliegenthart JFG. Human urokinase contains Ga1NA *c* β(1-4)[Fucα(1-3)]G1cNA *c* β(1-2) as a novel terminal element in *N* -linked carbohydrate chains. FEBS Lett. 1992;314:389–94. doi:10.1016/0014-5793(92)81512-K.

2. Vliegenthart JFG. The complexity of glycoprotein-derived glycans. Proc Japan Acad Ser B. 2017;93:64–86. doi:10.2183/pjab.93.005.

3. Neelamegham S, Aoki-Kinoshita K, Bolton E, Frank M, Lisacek F, Lütteke T, et al. Updates to the Symbol Nomenclature for Glycans guidelines. Glycobiology. 2019;29:620–4. doi:10.1093/glycob/cwz045.

4. Katoh T, Maeshibu T, Kikkawa K, Gotoh A, Tomabechi Y, Nakamura M, et al. Identification and characterization of a sulfoglycosidase from Bifidobacterium bifidum implicated in mucin glycan utilization. Biosci Biotechnol Biochem. 2017;81:2018–27. doi:10.1080/09168451.2017.1361810.

5. Rho J, Wright DP, Christie DL, Clinch K, Furneaux RH, Roberton AM. A Novel Mechanism for Desulfation of Mucin: Identification and Cloning of a Mucin-Desulfating Glycosidase (Sulfoglycosidase) from Prevotella Strain RS2. J Bacteriol. 2005;187:1543–51. doi:10.1128/JB.187.5.1543-1551.2005.
